# Charge and size effects in π-ligand activation: an IR spectroscopic study of gold–acetylene complexes

**DOI:** 10.1039/d5ra06762f

**Published:** 2025-11-13

**Authors:** J. Reichegger, M. Knabl, M. Schmidt, A. M. Reider, M. Ončák, P. Scheier, O. V. Lushchikova

**Affiliations:** a Institut für Ionenphysik und Angewandte Physik, Universität Innsbruck Technikerstr. 25 6020 Innsbruck Austria Johannes.Reichegger@uibk.ac.at

## Abstract

The electronic charge and size of metal clusters play a critical role in determining ligand activation, which is a key step in many catalytic processes. Here, the charge- and size-dependent interaction of gold clusters (Au_*n*_^+/−^, *n* ≤ 4) with up to four acetylene (C_2_H_2_) molecules is investigated using infrared photodissociation spectroscopy of He-tagged species, probing the C–H stretching region (2850–3390 cm^−1^). The IR spectra, supported by density functional theory calculations, reveal distinct trends in vibrational shifts, coordination geometries, and binding motifs that reflect the clusters' charge state and number of gold atoms. Cationic clusters activate acetylene *via* coordination bonds and π-backdonation. Gold cations up to *n* = 2 bind two acetylene ligands, while larger clusters coordinate only one. Additional molecules solvate the core cation, forming a second solvation shell. As the cluster grows, charge becomes increasingly delocalized across the ion-molecule complexes, which leads to a decrease in coordination number, weaker binding energy, and reduced acetylene activation. In contrast, anionic clusters interact only through polarization forces and quadrupole interactions, which do not lead to activation. These findings provide molecular-level insight into charge-controlled π-ligand activation and offer design principles for tailoring reactivity in charged metal complexes.

## Abstract 2

C_2_H_2_ behaves differently depending on the charge and size of gold clusters. Cations form coordination bonds, activating C_2_H_2_, whereas anions don't. The interaction weakens as size or ligand number grows.

## Graphical abstract

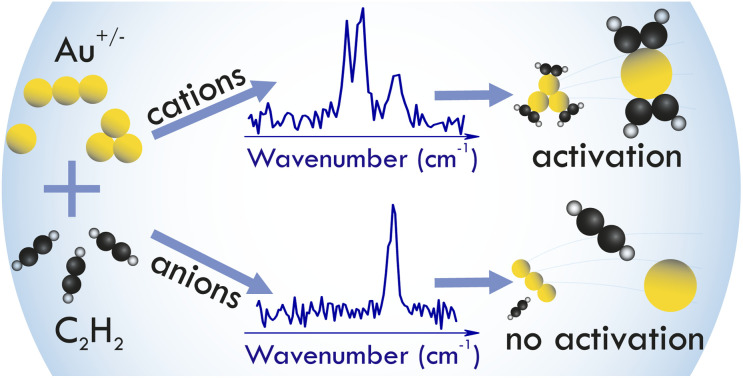

## 1 Introduction

Understanding how electronic charge influences ligand activation is essential for controlling chemical reactivity, particularly in catalysis. While charged metal clusters are frequently proposed as active species in supported catalysts or gas-phase reactions, the precise role of the charge state in defining the interaction strength and mode with π-ligands remains elusive.

Acetylene (C_2_H_2_), with its linear geometry, quadrupole moment, and accessible π* orbital, is both a fundamental alkyne prototype and a versatile industrial building block.^1–5^ It offers valuable insight into π-bonding to metals,^3,4^ and plays a key role in the synthesis of chemicals like vinyl chloride and ethylene.^2,5^ Therefore, understanding the metal–acetylene interactions at the molecular level is essential for both catalyst design and fundamental mechanistic studies.

Gold, despite its closed-shell d^10^ configuration, displays rich and unexpected reactivity, particularly in nanoparticle form.^6,7^ Supported gold nanoparticles, especially below 3 nm, exhibit strong charge- and size-dependent reactivity in transformations such as acetylene hydrochlorination^8^ and hydrogenation.^9–11^ They outperform traditional catalysts in selectivity and have already replaced mercury-based systems in vinyl chloride production.^5,12^ Although less active than Pd in acetylene hydrogenation, their high selectivity makes them promising for future applications.^5,9,10,13^

Cationic species such as Au^+^ and Au_3_^+^ have been identified as key active sites,^5,14–17^ with their charge states influenced by the nature of the support. Oxide materials such as TiO_2_, CeO_2_, and SiO_2_ tend to stabilize gold in positive oxidation states.^11,13,17–19^ It was suggested that nitrogen- or boron-doped carbon supports can favor either cationic or anionic charge states.^20^ In this context, acetylene has been described as a “Janus” ligand, acting as an electron donor or acceptor depending on the metal's charge.

To eliminate support effects and isolate intrinsic interactions, we examine cationic and anionic gold clusters (Au_*n*_^+/−^, *n* ≤ 4) in the gas phase. Since metallic gold clusters are relatively inert, whereas charged species are catalytically active, this charge-controlled reactivity is central to understanding ligand activation.^5,14–17,20,21^

Previous density functional theory (DFT) studies primarily addressed neutral Au clusters, showing stronger C_2_H_2_ activation for odd-numbered clusters and better performance of smaller species.^22–25^ However, only a few studies have addressed the interaction of C_2_H_2_ with charged coinage metal clusters, and these were mostly limited to triatomic systems.^26^

Experimentally, gas-phase ionic metal–acetylene complexes have been characterized by mass spectrometry,^4,27^ as well as electronic,^28–32^ and infrared vibrational spectroscopy.^33–42^ These techniques have provided comprehensive information about their electronic and geometrical structures, fragmentation patterns, reactivity, and binding energies.

IR photodissociation spectroscopy, combined with DFT, is well suited to characterize ground state geometries. Duncan *et al.* found that monoatomic cations (*e.g.*, Mg, Ca, V, Fe, Ni, Cu, Zn, Ag, and Au) form T-shaped (η^2^) complexes,^33–42^ following the Dewar–Chatt–Duncanson (DCD) π-bonding model.^43,44^ According to this model, acetylene donates π-electrons into the metal's empty σ-type d orbital, while the metal's filled d orbital back-donates electrons into the π* antibonding orbital of acetylene. This interaction causes the linear acetylene molecule to bend, weakening both the C–C and C–H bonds. Consequently, the vibrational frequencies shift to lower values compared to the measured C–H stretching mode of free acetylene at 3288.7 cm^−1^.^45^

For complexes with first-row metal cations from V to Ni, the C–H frequency systematically redshifts, while coinage metals show weaker shifts, likely due to their filled d orbitals. Notably, Au binds more strongly than Cu or Ag due to relativistic effects.^34,35,41^ At higher ligand loadings, Ni, Fe, Co, and Ag form tetrahedral π-complexes,^33,34,39,40,42^ whereas Au and Cu favor threefold nearly-planar geometries.^35,41^ V induces acetylene cyclization,^38,42^ and Zn forms either *D*_3h_ symmetric (η^2^) or vinyl-like (η^1^) structures.^37^

While the solvation of metal anions with acetylene remains largely unexplored, halide anions (Cl, Br, I) have been thoroughly studied by Bieske *et al.* using IR spectroscopy, revealing similar redshifts of the C–H stretch.^46–49^ However, the origin of these shifts differs: unlike the T-shaped coordination in cationic complexes, acetylene binds end-on to anions due to dominant charge–quadrupole interactions.

In the present study, multiply-charged superfluid He nanodroplets (HNDs) are utilized to perform photodetachment spectroscopy of He-tagged Au_*n*_^+/−^(C_2_H_2_)_*m*_ complexes, with *n* and *m* ranging from 1 to 4. This approach enables the examination of cold He-tagged complexes with both positive and negative charges, minimizing band shifts due to tagging and enhancing spectral resolution. While Vilesov *et al.* applied similar methods to large silver-acetylene clusters,^50^ our work focuses on small, well-defined ionic clusters, offering molecular-level insight into charge-dependent binding and activation.

## 2 Methods

### 2.1 Experimental section

Superfluid helium nanodroplets with a temperature of 0.37 K and a mean droplet size of a few million He atoms^51^ are formed upon supersonic expansion of pressurized He gas (2.7 MPa stagnation pressure, 99.9999% purity, Messer Austria GmbH) through a micrometer-sized nozzle mounted onto a closed-cycle cryocooler, Sumitomo RDK-415D2, 9.0–9.7 K, around 5 µm orifice diameter, into vacuum. After passing through a skimmer (0.8 mm diameter), the HNDs cross an electron beam that multiply ionizes them *via* electron impact at an electron energy of around 60 eV for cations and 30 eV for anions. Each charge serves as a nucleation center for cluster growth.

The highly ionized HND beam then crosses a pick-up chamber where gold is sublimated from a resistively heated Shapal ceramic oven at a set oven power of 195 W. The sequentially picked-up gold atoms are attracted to the helium charge centers by ion-induced dipole interactions. The first arriving gold atom is ionized *via* charge transfer, which is highly exothermic due to the large difference in ionization energy between helium (IE = 24.6 eV ^52^) and gold (IE = 9.2 eV ^53^). Further pick-up of dopants leads to the growth of singly charged clusters at each charge center. The fast dissipation of energy into the surrounding helium matrix prevents fragmentation of excited complexes and results in the evaporation of helium atoms from the droplets.

In the next step, the gold-doped HNDs enter a second pick-up chamber filled with C_2_H_2_ (10^−7^ mbar, acetylene dissolved in acetone, Linde). The doped HNDs pick up C_2_H_2_, leading to the formation of ligand–metal complexes. To release the dopant cluster ions from the large helium droplets and make them accessible for mass spectrometry, the doped HNDs collide with a stainless-steel surface mounted inside the second pick-up chamber at normal incidence. During the splashing process,^54^ charge centers are recoiled by reflecting helium or shock fronts. However, most of the extracted ions remain solvated by a countable number of He atoms, typically small enough to be accessible by mass spectrometry.

The solvated ions are guided from the collision region into a time-of-flight (ToF) mass spectrometer (Tofwerk AG model HToF) by applying weak electrostatic fields. Mass spectra with an average resolution of 1600 m/Δ*m* are obtained. A tunable pulsed laser (EKSPLA NT277, up to 80 µJ pulse energy at 3000 nm, laser bandwidth < 10 cm^−1^) is used to perform IR photodissociation spectroscopy upon vibrational excitation of the helium-tagged metal–ligand complexes. The laser is calibrated using a wavemeter (SHR High-Resolution Wide-Range Wavelength Meter). The ToF (operation frequency 10 kHz) and the laser (repetition rate 1 kHz) are synchronized to allow a direct comparison of the mass spectra with and without laser irradiation.

Photon absorption leads to vibrational excitation of the Au_*n*_^+/−^(C_2_H_2_)_*m*_ complexes, causing the subsequent evaporation of He atoms from the clusters. Monitoring the depletion of the He_*i*_Au_*n*_^+/−^(C_2_H_2_)_*m*_ ion signal and the corresponding increase in the Au_*n*_^+/−^(C_2_H_2_)_*m*_ ion signal indicates photon absorption. Multiple absorption spectra are recorded for Au_*n*_^+/−^(C_2_H_2_)_*m*_ in the range of 2850 to 3400 cm^−1^, covering the C–H stretch region of C_2_H_2_. All absorption spectra are corrected for differences in laser intensity at various wavelengths, assuming a direct correlation between photon count and ion signal changes. The photon number is derived from the measured laser power using eqn (1), where *P*_laser_ is the laser power, *h* is the Planck's constant, *c* is the speed of light, and *ν* is the calibrated wavenumber in cm^−1^.1
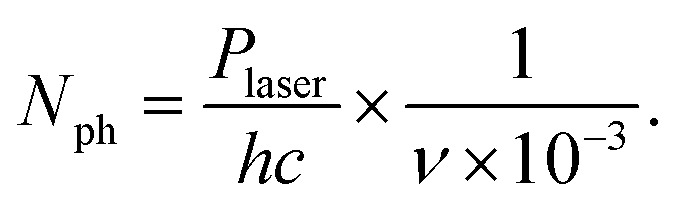


The raw data and laser power are measured simultaneously, enabling precise background and laser-power corrections. Since the repetition rate of the ToF is ten times higher than that of the laser, the signal is recorded in segments: one segment with the laser on (laser segment) followed by nine segments without the laser (dark segments). Each dark segment is analyzed across the entire mass range, from 30 u to 1850 u, as no depletion is expected in the absence of laser irradiation. The signal of each mass is averaged over all dark segments. The individual averages are then summed up over all masses to obtain the background signal, which is used to correct the raw signal according to eqn (2):2
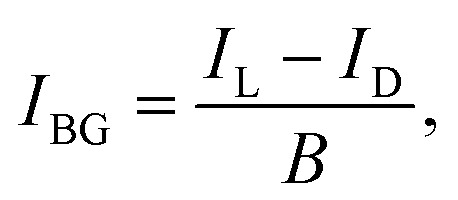
where the difference between the laser segment, *I*_L_, and the dark segment, *I*_D_, is normalized over the background signal, *B*. The background-corrected signal *I*_BG_ is further normalized to the number of photons, *N*_ph_, giving the laser corrected signal *I*_LC_ in eqn (3).3
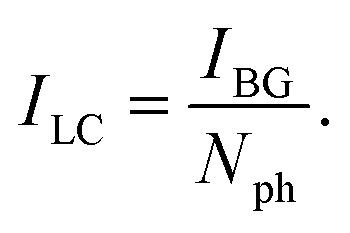


### 2.2 Computational section

Quantum chemical calculations are performed for Au_*n*_^+/−^(C_2_H_2_)_*m*_ clusters. The structures of these clusters are calculated using density functional theory (DFT) at the ωB97X-D/def2TZVP level, as implemented in the Gaussian16 package.^55^ Relativistic effects are partially accounted for through effective core potentials in the basis set.

Due to the sub-Kelvin temperature of the helium matrix, only the ground-state clusters are considered, as higher electronic and vibrational excitations are inaccessible under this conditions according to the Boltzmann distribution. However, several isomeric structures are identified for different complexes, which are energetically close to the most stable isomer predicted.

The empirical scaling factor of 0.9552 is applied to the calculated infrared absorption peak positions to account for approximations in the chosen functional and basis set, as suggested by the Computational Chemistry Comparison and Benchmark Database.^56^ This scaling factor was adapted from the next closest basis set, being the def2TZVPP basis set. The charge distributions and their impact on the spectral properties of the ion-molecule complexes, as well as their binding behavior are quantified by calculating the electrostatic potential (ESP) charges, using the CHELPG method.^57^ For this purpose, the radius of gold atoms was defined as 1.66 Å. In addition, a natural bond orbital analysis was performed to further study the charge transfer within gold–acetylene complexes. The binding energies of C_2_H_2_ are investigated as a function of gold cluster size and the number of C_2_H_2_ ligands. Binding energies, *E*_B_, are calculated for the ligand elimination reaction according to eqn (4)

4 *E*_B_ = *E*_prod._ − *E*_react._,

where *E*_prod._ is the sum of the total energy of the reaction products and *E*_react._ the total energy of the reactants. In the case of this work, the intact metal–ligand complex is the reactant that dissociates into two product fragments

5 Au_*n*_^+/−^(C_2_H_2_)_*m*_ → Au_*n*_^+/−^(C_2_H_2_)_*m*−1_ + C_2_H_2_

All reported binding energies are corrected for zero-point energy contributions and basis set superposition errors.

As a final characterization of the gold–acetylene complexes, an energy decomposition analysis (EDA-NOCV), implemented within ORCA,^58^ was performed to identify the most contributing energy components with respect to the net charge and cluster size.

## 3 Results and discussion

### 3.1 Cations

**Fig. 1:**
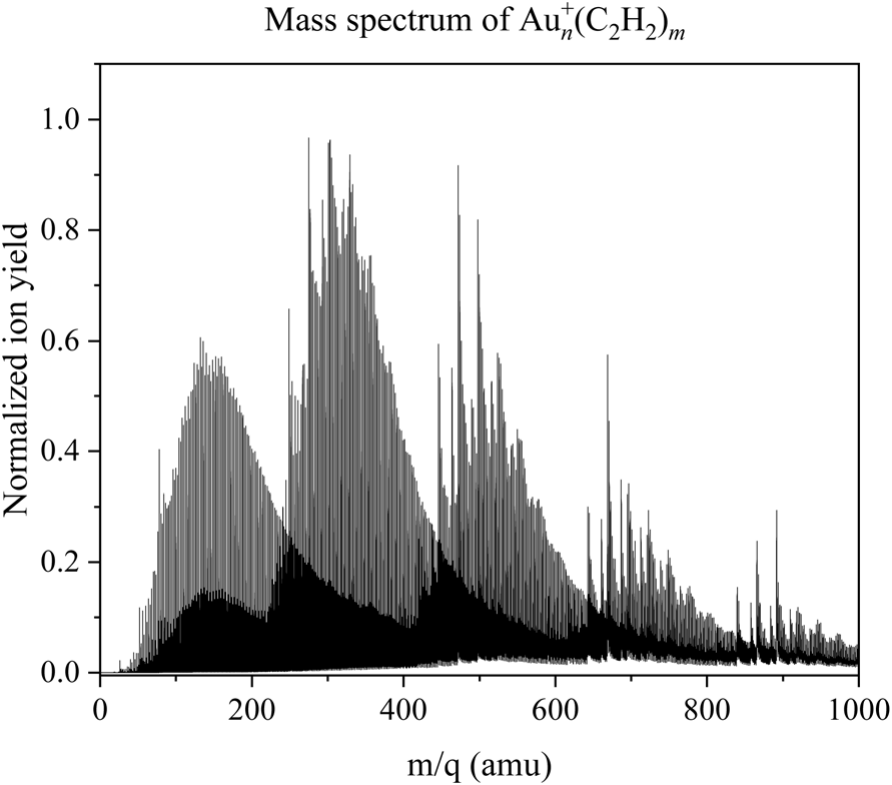
Mass spectrum of cationic gold–acetylene clusters in the range of 1 amu to 1000 amu. Up until the gold monomer with mass 197 amu the major contributions are pure helium clusters and acetylene. Afterwards the gold–acetylene clusters emerge; four distinct mass distributions are visible. The ion yield and the amount of attached helium decrease with increasing cluster size.

**Fig. 2:**
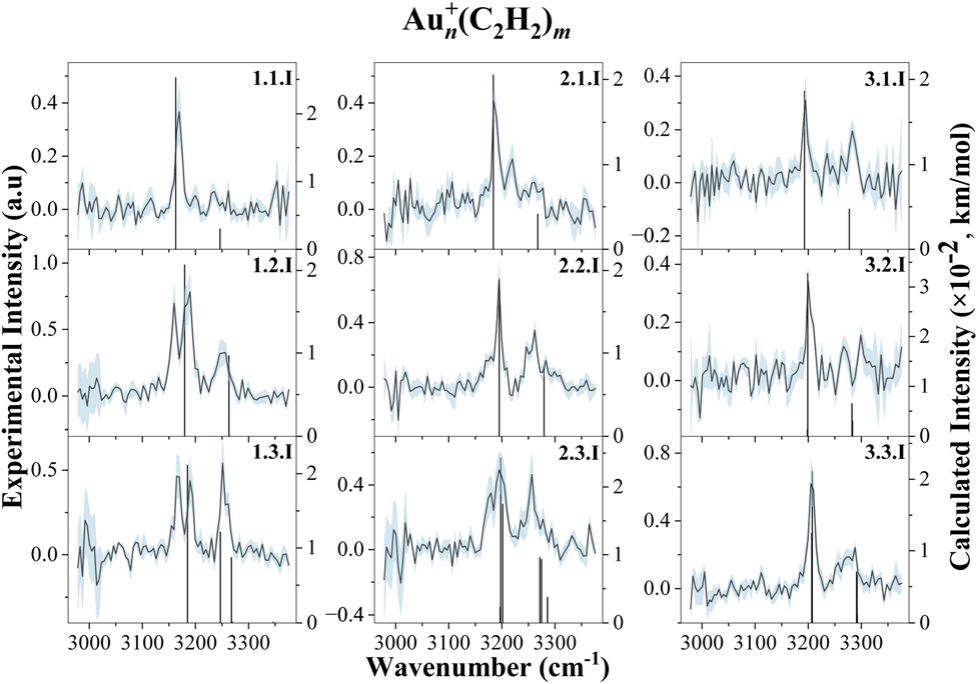
Experimental spectra of Au_*n*_^+^(C_2_H_2_)_*m*_. The dark traces, corresponding to the experimental data, are embedded within the statistical uncertainties in blue. The black vertical lines represent the calculated vibrational transitions. The calculated intensities are multiplied by a factor of 10^−2^. The columns represent the size of the gold cluster *n* = 1–3, while the rows indicate the number of acetylene ligands on the gold cluster *m* = 1–3. Each spectrum is labeled by the number of gold atoms *n*, the number of acetylene ligands *m* and a roman numeral for the respective isomer.

IR spectra of Au_*n*_^+^(C_2_H_2_)_*m*_ were obtained in the range of 2985 to 3390 cm^−1^, covering the C–H stretch region of acetylene, for all combinations of *n*, *m* = 1–3, as shown in Fig. 2, and for *n*, *m* = 4, as illustrated in Fig. 4. For (*n* = 4, *m* = 1–3) and (*n* = 1–3, *m* = 4), the signal intensity was insufficient, due to a combination of low ion yield and weak helium solvation for these particular cluster complexes (see Fig. 1). Most spectra exhibit two bands with an average bandwidth (full width at half maximum, FWHM) of around 10 cm^−1^, which shift to higher wavenumbers with increasing cluster size and number of C_2_H_2_ molecules, indicating progressive weakening of metal–ligand interactions.

**Fig. 3:**
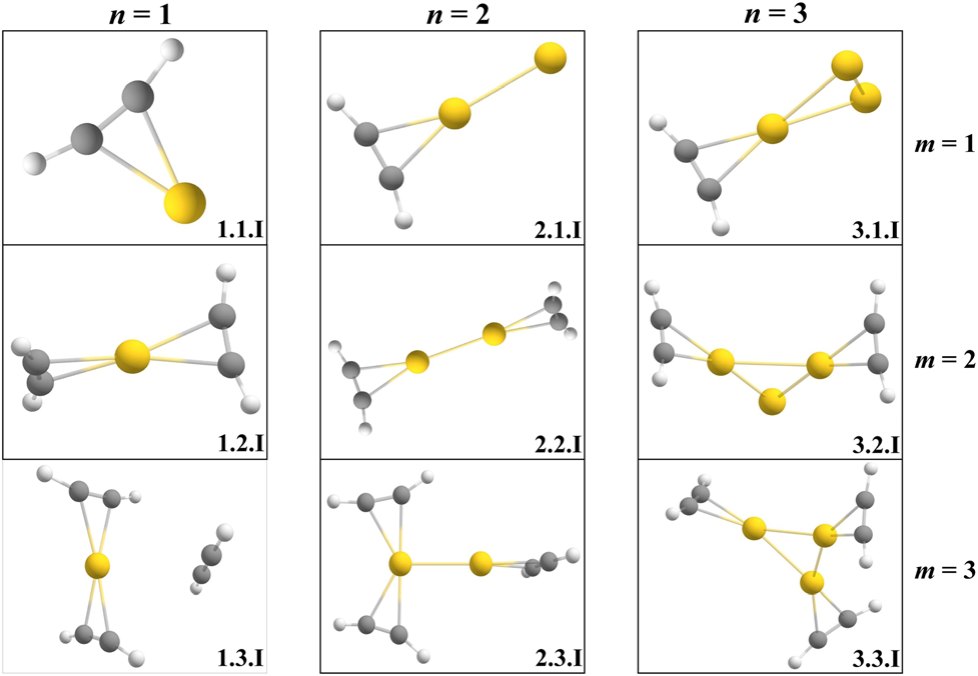
Calculated isomers of Au_*n*_^+^(C_2_H_2_)_*m*_ that correspond to the IR spectra shown in Fig. 2.

**Fig. 4:**
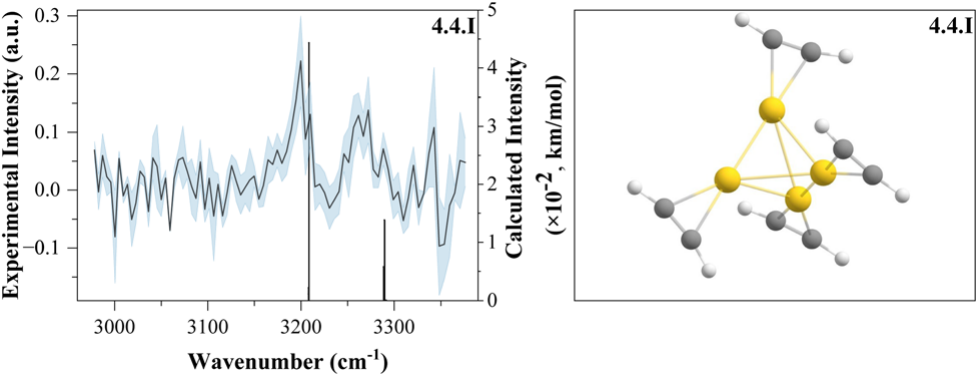
The spectrum of Au_4_^+^(C_2_H_2_)_4_ (left) along with the calculated isomer (right). The calculated intensities are multiplied by a factor of 10^−2^.

To gain insight into cluster structure and properties, the experimental spectra were compared to DFT calculations at the ωB97X-D/def2TZVP level of theory. The computed spectra that show the best agreement with the experimental data are shown in Fig. 2 and 4 (left), along with the corresponding structures, displayed in Fig. 3 and 4 (right). A detailed list of all experimental measured and calculated C–H stretch frequencies and their assignment can be seen in Table 1 of the SI. Further calculated isomers of the cations and anions are included in Fig. S3 and S4 of the SI.

The interaction of acetylene (*m* = 1–3) with a monoatomic gold cation (*n* = 1) gives rise to up to three absorption bands in the 3150 cm^−1^ and 3300 cm^−1^ region, red-shifted relative to the two antisymmetric stretch bands of free C_2_H_2_ measured at 3294.9 cm^−1^ and 3281.9 cm^−1^, which arise from a Fermi resonance between the C–H stretch and a combination of the C≡C stretch and overtone of the bending vibrations, and the symmetric stretch band of free acetylene measured at 3374 cm^−1^.^59^ The observed red shifts suggest a weakening of the C–H bond and indicate activation of the acetylene molecules upon binding, deviating from the linear geometry of free acetylene. DFT calculations suggest that all ligands adopt an η^2^ (T-shaped) geometry and are significantly bent. This bending is indicative of strong π-interaction with the metal center, leading to the formation of coordination bonds followed by the activation of acetylene molecules as previously reported by Duncan and co-workers.^41^ Because of the interaction with the metal center, molecular symmetry is broken, allowing the otherwise IR-inactive symmetric stretch to become observable. However, for *m* = 1, the symmetric stretch is not resolved experimentally, likely due to low photodissociation yield at the available laser power. Interestingly, this symmetric mode emerges clearly in the spectra upon the addition of a second or third ligand, or with increasing cluster size, suggesting a correlation between activation and complex geometry.

In the case of *m* = 1 and *m* = 2, the band corresponding to the antisymmetric stretch appears at 3169 cm^−1^ and 3183 cm^−1^, respectively, while the symmetric stretch becomes visible only for *m* = 2 at 3250 cm^−1^. The predicted structures for these complexes align well with structures previously reported by the Duncan group.^41^ However, the IR spectrum for *m* = 2 also exhibits an unexpected feature, a relatively intense band at 3160 cm^−1^. This band may result from the elimination of one acetylene from the Au^+^(C_2_H_2_)_3_ complex, leading to Au^+^(C_2_H_2_)_2_ as a photofragment. The previous study shows that every additional acetylene *n* > 2 is weakly bound to the central metal ion and can form a second solvation shell.^41^ According to energetic calculations, the third acetylene ligand is only weakly bound by 0.27 eV, corresponding to 2177 cm^−1^. Because there is no mass selection before the laser irradiation, all possible metal–ligand dissociation channels are simultaneously accessible. As a result, multiple dissociation pathways from different parent ions could lead to the same photofragment, which is subsequently detected by the ToF.

For *m* = 3, coordination saturation appears to be reached, and a transition toward a second solvation shell occurs. In contrast to *m* = 1 and *m* = 2, the third acetylene is bound to the Au^+^ through ion-induced dipole forces, consistent with the weak binding energy. The antisymmetric stretch band is found at 3190 cm^−1^, while the symmetric region displays a splitting: an intense peak at 3250 cm^−1^ and a shoulder at 3259 cm^−1^. This splitting in the experimental spectrum is consistent with the theoretically predicted 2C+1 structure, in which two ligands are directly coordinated, while the third one is weakly attached. The band at 3250 cm^−1^ can be identified as the antisymmetric stretch motion of the nearly free acetylene molecule, while the 3259 cm^−1^ peak corresponds to the symmetric stretch of the aforementioned activated bound ligands.

In addition to the most stable 2C+1 isomer, two alternative 3C isomers were found that have the third acetylene bound to the central gold atom. Energetically, the 3C isomers are predicted to lie just 31 meV and 34 meV higher. The first isomer has one of the three acetylene molecules rotated orthogonally to the plane that goes through the atomic centers of all the other atoms. The second isomer adopts a complete planar structure as reported by Duncan *et al.*^41^ Contrary to their work, the lower lying 2C+1 configuration shows the best agreement with the experimental spectrum. This discrepancy may be attributed to two facts. First, the experimental spectra are recorded by helium tagging and thus below a few Kelvin, in contrast to Ar tagging and room temperature experiments.^60,61^ Secondly, DFT results may vary depending on the choice of functional and basis set, introducing uncertainty in the isomer energetics. Additionally, our IR spectrum shows yet another band at 3168 cm^−1^, which likely originates from acetylene elimination from the Au^+^(C_2_H_2_)_4_ complex, as previously explained.

The results for the cationic gold atom point towards a coordination number of two for acetylene binding. However due to the uncertainty in the DFT calculations for Au^+^(C_2_H_2_)_3_ and its three isomers and the difficulty to distinguish them based on the experimental results in this frequency range, the coordination number for the cationic gold atom could also be three. From this point onward, the coordination number will be defined as the number of ligands that bind per gold atom.

**Fig. 5:**
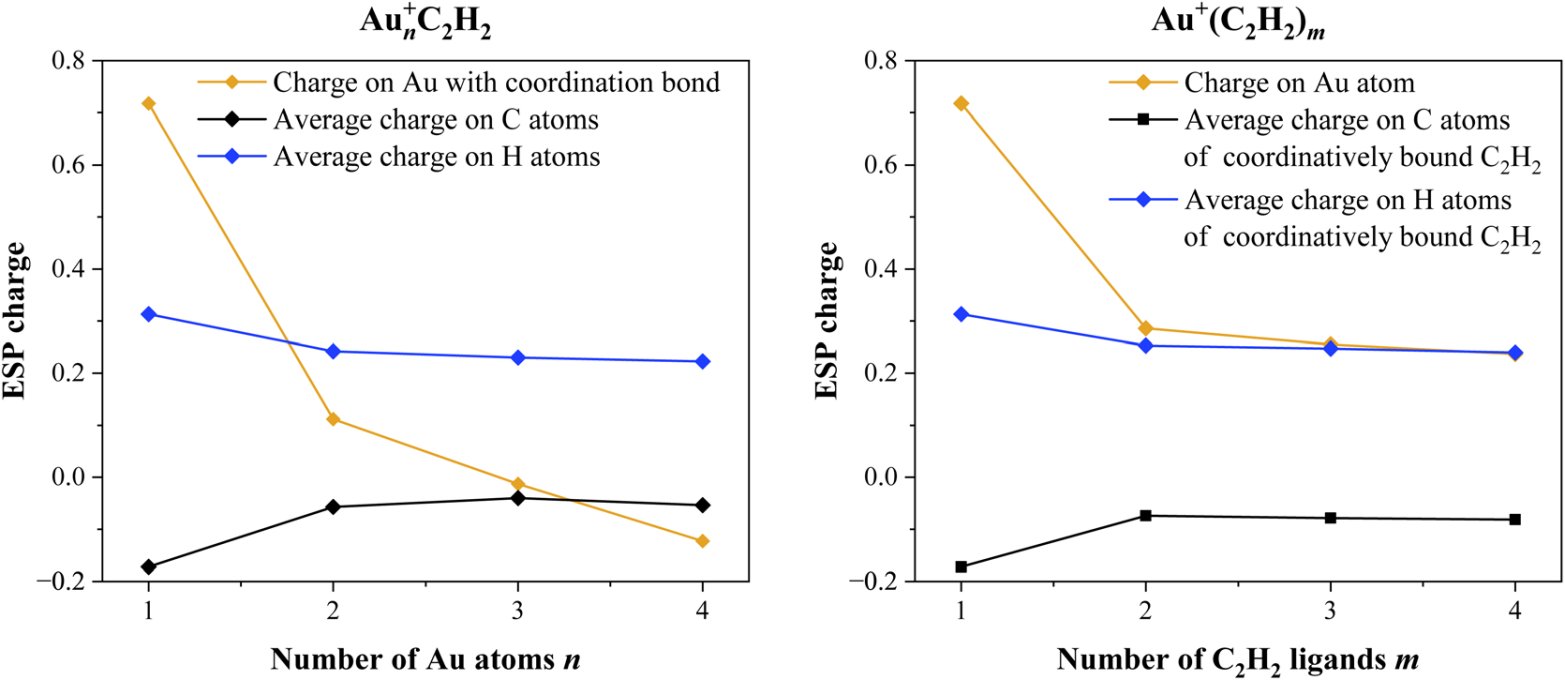
Left: CHELPG charges as a function of the number of gold atoms *n*. Only the charge on the gold atom that is involved in the coordination bond to C_2_H_2_ is depicted. Right: CHELPG charges as a function of the number of C_2_H_2_ ligands *m*. In both cases, an average charge is given to the C and H atoms, since their values are in a similar range.

As the number of attached C_2_H_2_ molecules increases, the vibrational bands in the spectra are blue-shifted, suggesting a progressive weakening of the metal–ligand interaction. This shift reflects reduced charge transfer from the gold cation to the acetylene ligands as the coordination shell becomes saturated, resulting in less activation of the C–H bonds. To investigate this effect quantitatively, atomic charges were calculated for the gold monomer with up to four acetylene ligands, as well as for gold clusters up to the tetramer, all with one acetylene ligand attached (see Fig. 5). This method allowed to observe the general trend for the charge transfer along *n* and *m*.

The results of these calculations showed that the positive charge is increasingly delocalized over the gold atoms in larger clusters, resulting in the lower partial charge on bound gold atoms. Additionally, polarization effects start to play a role on larger gold clusters because of their metallic character. This becomes especially apparent for the gold tetramer, where the partial charge on the gold atom with the coordination bond is slightly negative. However, accompanying NBO calculations do not attribute a negative charge to this particular gold atom. Therefore, we concluded that it could be an artifact of the CHELPG calculations. The overall trends however remain the same. Moreover, the positive charge is further reduced with each additional C_2_H_2_ ligand forming a coordination bond. However, ligands occupying the second solvation shell induce only marginal changes in the charge distribution on the gold atoms, consistent with a weaker, polarization-based interaction. This finding supports the experimental observation that only ligands in the first solvation shell are activated, while additional ligands contribute minimally to the overall charge redistribution and vibrational perturbation. Thus only ligands in direct coordination significantly alter the electronic structure of the metal center. To further underline all these points, natural bond orbital (NBO) analysis was carried out. The natural electron configurations in respect to gold cluster growth and acetylene ligand attachment are listed in Section 4 of the SI. The results of these calculations are in good agreement with the aforementioned CHELPG results.

With the addition of a second gold atom (*n* = 2), two main absorption bands are observed in the experimental spectrum between 3150 cm^−1^ and 3300 cm^−1^, corresponding to the symmetric and antisymmetric C–H stretching modes of the coordinated acetylene molecule. The gold dimer can bind two acetylene molecules per gold atom before a second solvation shell is populated by the ligands. Interestingly, the metallic bond between the two gold atoms does not significantly alter the primary coordination number, which remains two with respect to acetylene binding. For *m* = 1, the position of the antisymmetric stretch band is at 3189 cm^−1^, shifting slightly to 3194 cm^−1^ and 3196 cm^−1^ for *m* = 2 and 3, respectively. The symmetric stretch, again weak or absent at low coordination for *m* = 1, becomes visible at 3259 cm^−1^ for *m* = 2 and gets strong at 3256 cm^−1^ for *m* = 3. Band splitting is observed for *m* = 3, with an additional peak emerging at 3177 cm^−1^. The energy needed for acetylene elimination is insufficient in the IR wavelength range the laser is operated in. Moreover, DFT calculations do not predict any isomeric structures that would have such a vibration. These two arguments rather suggest that this peak might be a part of the overall noise.

The gold trimer (*n* = 3) shows two primary absorption bands in the IR spectrum, though a decreasing signal-to-noise (S/N) ratio becomes already noticeable for *m* = 1 and 2. The peak at 3194 cm^−1^ for *m* = 1 is assigned to the antisymmetric stretch of C_2_H_2_, shifting to 3201 cm^−1^ and 3207 cm^−1^ for *m* = 2 and *m* = 3, respectively. The symmetric stretch vibration is only confidently identified for *m* = 3 at around 3279 cm^−1^.

The S/N ratio becomes too low to reliably extract spectra for *n* > 3. However, a spectrum for *m* = 4 is observed when four C_2_H_2_ ligands are bound to the cluster. Two vague absorption bands were found around 3197 cm^−1^ and 3263 cm^−1^, corresponding to the antisymmetric and symmetric stretch vibration. The additional gold atom provides another binding center for C_2_H_2_. Thus all C_2_H_2_ ligands are coordinated, unlike in *n* = 3, where *m* = 4 will form a new weakly bound solvation shell. However, it is interesting to note that the gold tetramer can be found in a planar 2D structure or in a tetrahedral 3D structure. Typically, free gold clusters are found in a planar structure.^62^ We observe both; the tetrahedral structure appears for *m* > 1 and happens to be the more energetically favorable structure, lying Δ*E* = 0.06 eV below the planar structure on average. Within the scope of this experiment, no definitive conclusion regarding the gold cluster structure can be drawn, as only the C–H stretching region was measured. Structures are assigned purely through DFT calculations.

The results suggest that the coordination number with respect to acetylene binding decreased from two to one when *n* > 2. All acetylene molecules that form a coordination bond remain activated as indicated by red-shifted and split IR absorption bands compared to the free C_2_H_2_. Although the experimental spectra are limited by a declining signal-to-noise ratio for larger clusters, theoretical calculations considered metal–ligand complexes up to *m* = 5. These calculations indicate that each gold atom preferentially binds only one acetylene molecule in the first solvation shell for *n* > 2. Additional acetylene ligands are bound more weakly and are interpreted to form the second solvation shell.

**Fig. 6:**
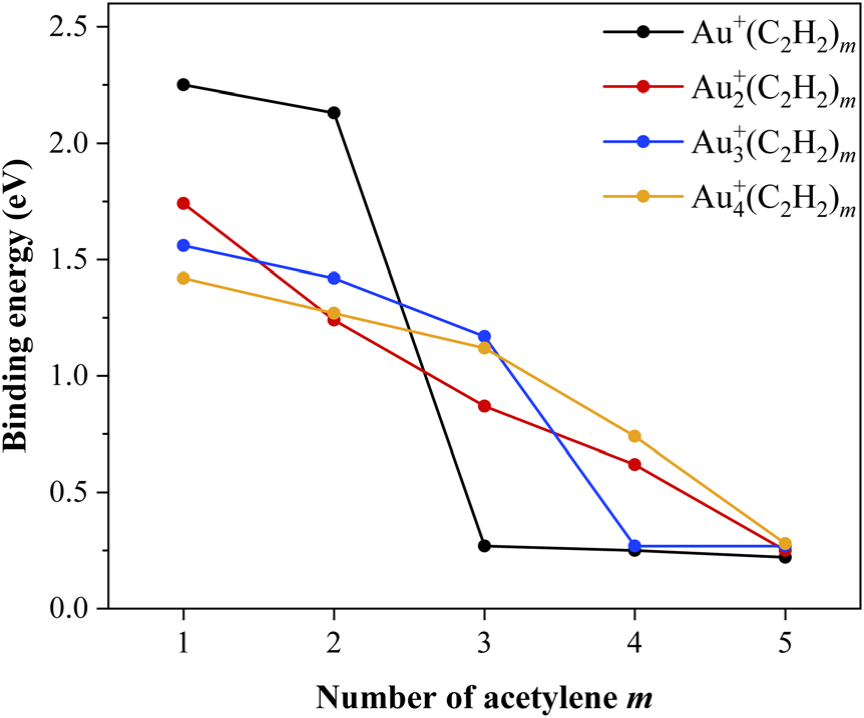
Binding energies of the individual acetylene molecules to each gold cluster. The coordination number per gold atom is two for the monomer and dimer and transitions to one for the trimer and tetramer. The coordination number can be identified from the point where the energy drops to around 0.25 eV, which signals the formation of the second solvation shell.

This behavior, where each C_2_H_2_ molecule occupies its own gold atom, is also reflected in the binding energy trends, shown in Fig. 6. The monomer binds its first two acetylene ligands with an energy around 2.2 eV, followed by a steep drop to around 0.25 eV for the third C_2_H_2_ molecule. Each subsequent C_2_H_2_ molecule binds with similarly low energy. The gold dimer shows binding energies, ranging between 0.6 eV and 1.7 eV for *m* = 1 − 4, with a pronounced decrease to around 0.25 eV at *m* = 5. Even though each gold atom binds two acetylene ligands with a coordination bond, the influence of the Au–Au metallic bond on the overall coordination environment becomes evident. The energy difference between the fourth and fifth acetylene molecule gets smaller compared to the steep drop in the case of the gold monomer. Another consequence of the gold cluster growth is the emergence of steric effects that will start to dictate how many ligands can bind per gold atom. Larger gold clusters have less room available for multiple C_2_H_2_ ligands to bind to the same gold atom without repulsion or geometric strain.

For the gold trimer, the coordination number per gold atom has fully transitioned from two to one. Three acetylene ligands bind *via* coordination–covalent interactions with an energy ranging between 1.2 eV and 1.6 eV, after which the binding energy drops to around 0.25 eV for each additional ligand. In the case of the tetramer, the trend is again more gradual. The binding energy for the four covalently bound acetylene molecules ranges between 0.7 eV and 1.4 eV, while a decrease to approximately 0.25 eV occurs for the fifth.

The local energy decomposition analysis is in line with previous results. The electrostatic energy, the orbital energy, the Pauli energy and the exchange energy components are strong for coordinated acetylene molecules and decrease significantly for the uncoordinated ligands. The dispersion energy component remains weak through all cluster sizes. A detailed overview of all energies is given in Section 6 of the SI.

These results illustrate that ligand activation is highly sensitive to charge localization, which decreases with increasing cluster size. While small clusters can support multiple activated acetylene ligands *via* strong η^2^ interactions, larger cations shift to single-site coordination with weaker activation. This trend reflects both electronic saturation and growing charge delocalization.

### 3.2 Anions

**Fig. 7:**
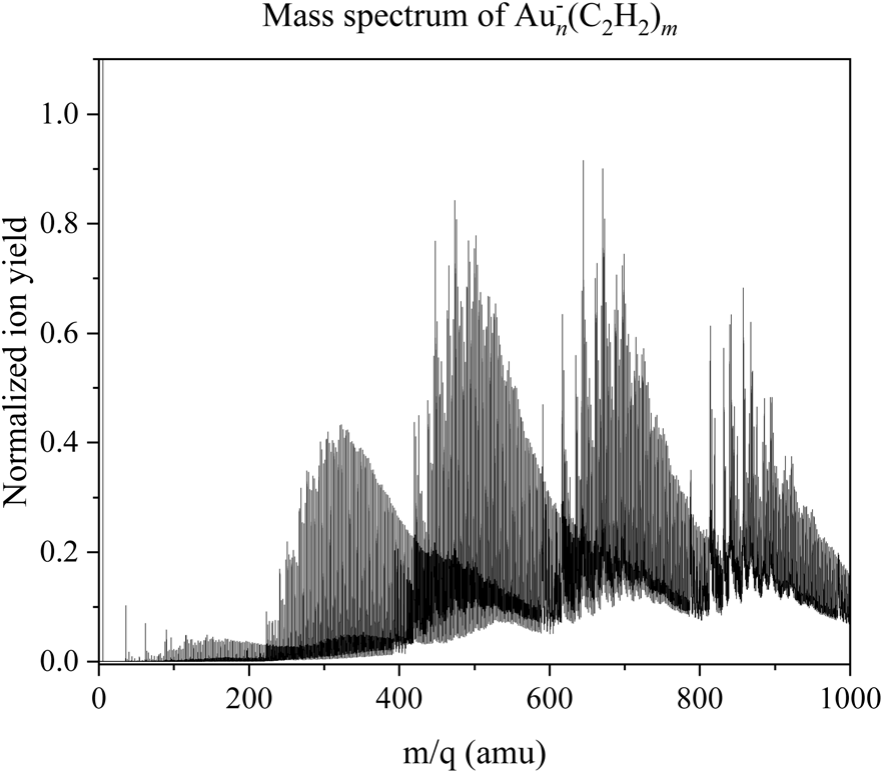
Mass spectrum of anionic gold–acetylene clusters in the range of 1 amu to 1000 amu. Up until the gold monomer with mass 197 amu the major contributions are pure helium and acetylene clusters. Afterwards the gold–acetylene clusters emerge; four distinct mass distributions are visible. The ion yield and the amount of attached helium decrease with increasing cluster size.

**Fig. 8:**
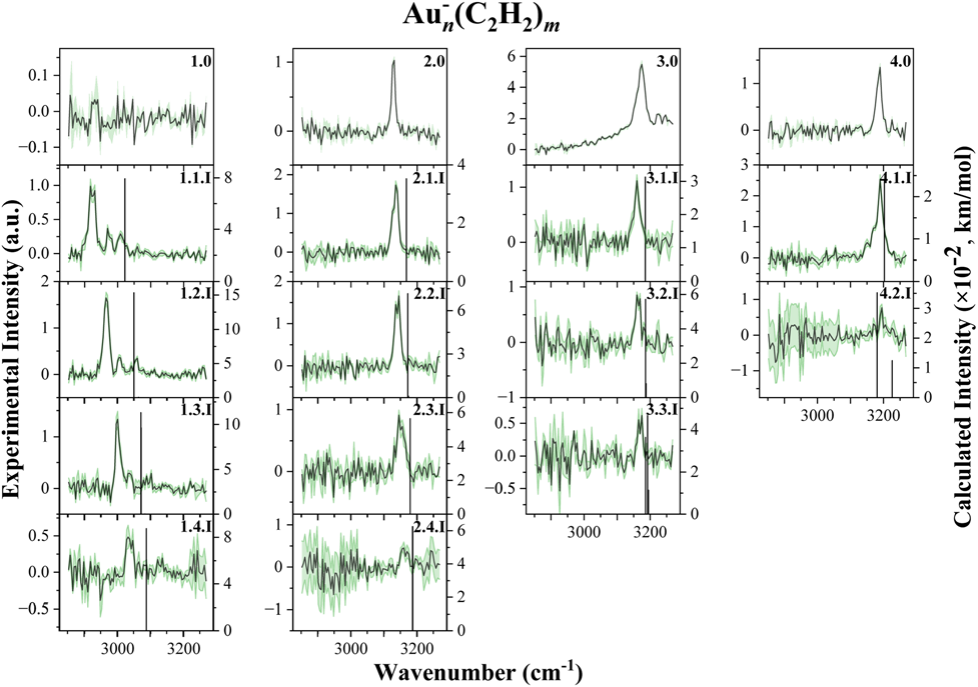
Experimental spectra of Au_*n*_^−^(C_2_H_2_)_*m*_. The dark traces, corresponding to the experimental data, are embedded within the statistical uncertainties in green. The black vertical lines represent the calculated vibrations. The calculated intensities are multiplied by a factor of 10^−2^. The columns represent the size of the gold cluster *n* = 1–4, while the rows indicate the number of acetylene ligands on the gold cluster *m* = 0−4. Each spectrum is labeled by the number of gold atoms *n*, the number of acetylene ligands *m* and a roman numeral for the respective isomer.

**Fig. 9:**
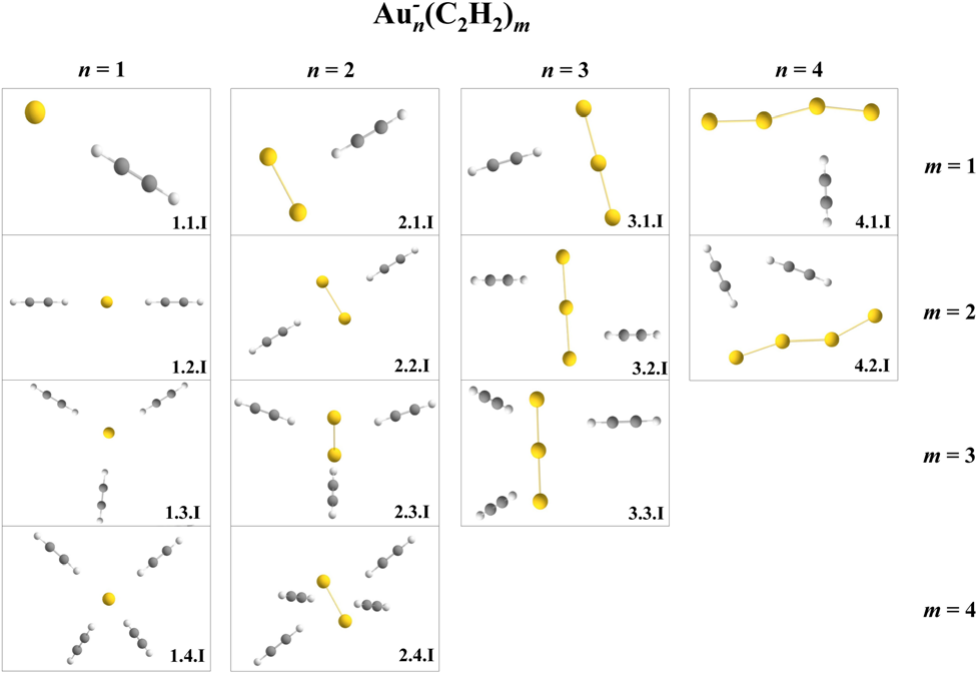
Calculated isomers of Au_*n*_^−^(C_2_H_2_)_*m*_ that correspond to the IR spectra shown in Fig. 8.

In the case of anions, IR spectra were recorded between 2850 and 3270 cm^−1^ for combinations of *n*, *m* = 0–4, except for (*n*,*m*) = (3,4),(4,3),(4,4), where the ion yield was insufficient. A corresponding mass spectrum is shown in Fig. 7. Most spectra show one principal absorption band, typically around 20 cm^−1^ FWHM, similar to the halides studied by the group of Bieske.^46–49^ This broadening might originate from unresolved absorption features or weaker metal–ligand binding interaction. DFT calculations (ωB97X-D/def2TZVP) were carried out to gain further insight into the structures and binding motifs of the anionic complexes. Fig. 8 includes experimental spectra and the computed spectra with the best agreement. Corresponding structures are shown in Fig. 9.

The monomeric anion Au^−^C_2_H_2_ shows a pronounced blue shift upon sequential addition of ligands, similar to the work of Bieske *et al.*^46–49^ This trend largely vanishes for larger clusters. In contrast to the cationic series, anionic gold clusters do not form coordination bonds with C_2_H_2_. The additional electron in the 6s orbital of gold hinders the π electron donation from the acetylene molecule, preventing the formation of η^2^-type complexes. Hence, the metal clusters and acetylene ligands interaction is dominated by polarization forces and acetylene molecules bind in an end-on configuration to the central anionic gold cluster. As a consequence, acetylene is not activated. CHELPG charge analysis and NBO analysis confirms that Au^−^ and C_2_H_2_ exhibit charge–quadrupole interaction.

Knowing the nature of the binding between gold anions and acetylene, the interpretation of the experimentally observed bands proves to be easier than that for the cations. In all measured anionic complexes, the dominant IR absorption can be assigned to the antisymmetric C–H stretch of the unperturbed acetylene molecule. In the case of anions, the symmetric stretch vibration remains IR-inactive, consistent with linear, non-activated binding geometries. Since the molecular symmetry is preserved, no mode mixing or symmetry breaking occurs, and no additional vibrational bands from activated ligands are observed.

**Fig. 10:**
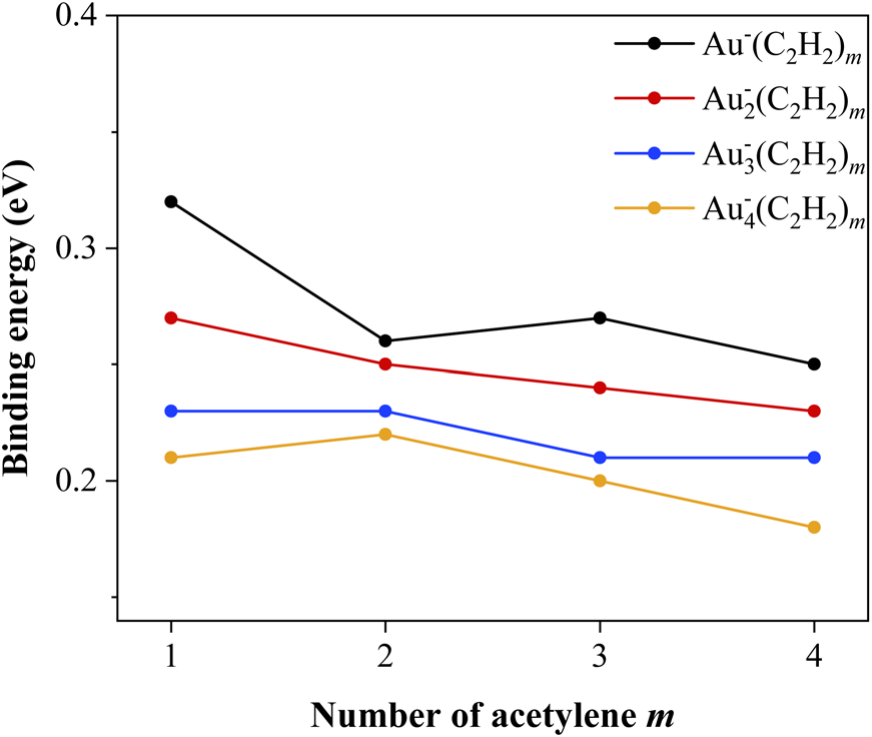
Binding energies of the individual acetylene molecules to each gold cluster. Each acetylene molecule binds similarly low to the gold cluster as the acetylene molecules in the second solvation shell of the cations. The growth of the gold cluster and therefore an increased delocalization of the charge has only a minor influence on the binding energy of acetylene.

IR spectra for *m* = 0 were also recorded and should not be interpreted as vibrational features of bare gold anions. Rather, these bands arise from photodissociation of weakly bound Au_*n*_^−^(C_2_H_2_)_*m*_ complexes. A look at the dissociation energies of the anionic cluster complexes in Fig. 10 reveals that they are at a level similar to the energy of the acetylene molecules in the second solvation shell of the cations. The dissociation threshold of the Au^−^C_2_H_2_ metal–ligand complex is 0.32 eV of energy, which corresponds to a laser energy of roughly 2581 cm^−1^. Operating the laser between 2850 and 3270 cm^−1^ should therefore dissociate these cluster complexes. Hence photodissociation takes place, followed by an increased ion yield of pure gold anions, which are detected as a photofragment at particular wavelengths.

Another interesting observation is the appearance of multiple bands within the gold monomer spectra. According to calculations, only one strong vibration should be observed. Further comparison of peak positions across the spectra reveals that many bands appear at similar wavenumbers, independent of cluster size or ligand number. This consistency suggests that the signals likely result from photodissociation of the C_2_H_2_ ligand itself, rather than the helium tag. The weak interaction between acetylene and the anionic gold clusters makes ligand loss more favorable, supporting this interpretation.

**Fig. 11:**
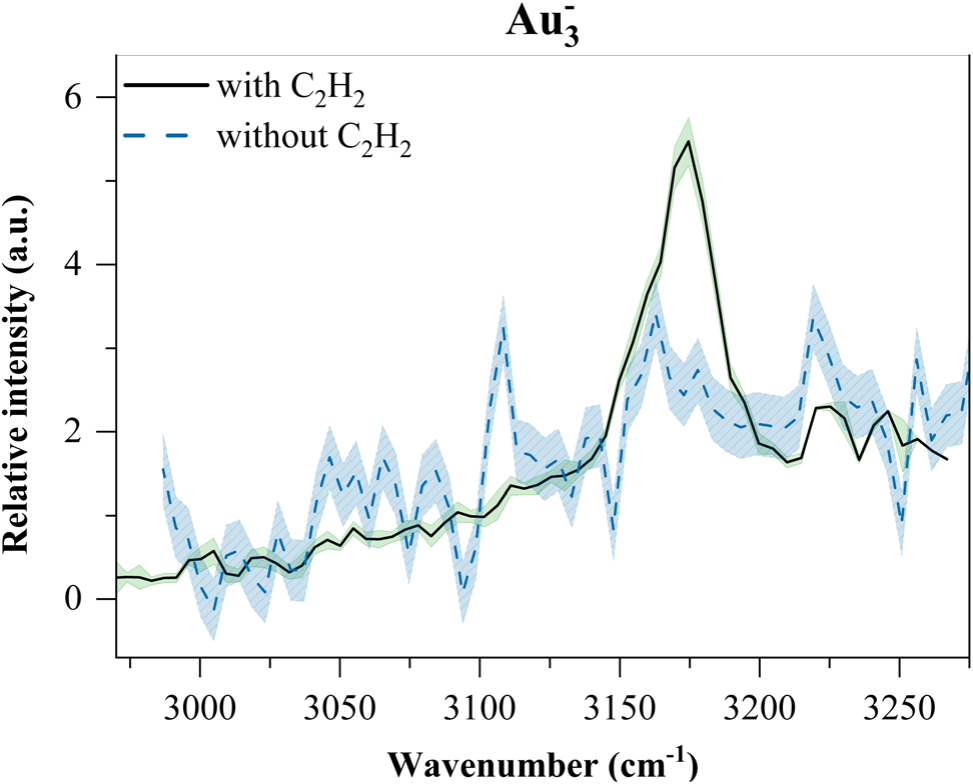
Spectrum of Au_3_^−^(C_2_H_2_) with a spectrum of pure Au_3_^−^ overlaid for comparison.

One unusual feature appears in the IR spectrum of Au_3_^−^: a broad, underlying absorption, which is present even in the absence of acetylene pickup (see Fig. 11). The feature persists across multiple conditions and is independent of ligand binding. Its origin remains unclear and may relate to unique low-frequency or electronic modes of the Au_3_^−^ cluster. Further investigation is required.

## 4 Conclusion

Cationic and anionic gold–acetylene ion-molecule complexes, Au_*n*_^+/−^(C_2_H_2_)_*m*_ (*n* ≤ 4, *m* ≤ 5), were investigated with respect to their coordination, structure, binding interaction and charge distribution at low temperatures. These studies were carried out using mass spectrometry and IR photodissociation spectroscopy of helium-tagged metal–ligand complexes in the C–H stretch region. The experiment was complemented by DFT calculations at the ωB97X-D/def2TZVP level of theory. The combination of experimental spectra and theory revealed that acetylene ligands form coordination bonds with cationic gold clusters. Most spectra show two main IR bands, corresponding to the symmetric and antisymmetric C–H stretching modes. Both bands are red-shifted compared to free acetylene as a result of π electron donation to the gold cation and subsequent activation of acetylene. The magnitude of this red shift decreases with increasing gold cluster size *n* and ligand number *m*, due to charge delocalization across the complex. The charge distributions were analyzed using CHELPG charges and natural bond orbital analysis. In general, all coordinated C_2_H_2_ ligands are activated and adopt an η^2^ (T-shaped) coordination geometry. The coordination number is two for Au_*n*_^+^(C_2_H_2_)_*m*_ for *n* ≤ 2 and decreases to one for larger clusters *n* > 2. Additional C_2_H_2_ molecules are bound through weaker polarization forces, forming a second solvation shell. This coordination trend is also reflected in the calculated binding energies, which decrease with increasing cluster size, due to charge delocalization. The binding energy of the acetylene molecules within the second solvation shell is around 0.25 eV across all gold cluster sizes, providing a benchmark for polarization-driven interactions.

In contrast, the interaction of anionic gold clusters with C_2_H_2_ is purely governed by polarization forces. This interaction is so weak that photodissociation of free acetylene itself is observed. No evidence of coordination bonds forming or ligand activation was found. Charge distribution calculations show that the interaction is primarily a charge–quadrupole effect, which becomes weaker with increasing gold cluster size and the number of ligands, due to charge delocalization.

These findings elucidate the fundamental role of charge state and cluster size in determining the electronic structure and reactivity of metal–ligand systems. The study underscores the utility of cold-ion spectroscopy in resolving fine structure and bonding motifs, and suggests that only cationic gold clusters may serve as viable candidates for gas-phase ligand activation. Future studies employing mass-selected photodissociation will allow more precise assignments of fragment channels and deepen our understanding of charge-dependent reactivity at the molecular level.

## Author contributions

JR has done the experimental and computational investigation, formal analysis and data curation, as well as the validation and visualization. He has written the original draft and is part of the review & editing. MK was involved in the experimental investigation, formal analysis, data curation and validation. He was also part of the visualization. MS has done the experimental investigation and validation, developed software and was involved in the review. AMR was involved in the experimental investigation and validation. MO provided resources and supervised the computational side of this work. He validated computational results and reviewed and edited this manuscript. PS supervised the experimental side of this work. He has done the funding acquisition, provided resources, developed the methodology and has reviewed and edited this manuscript. OVL has done the conceptualization and project administration and supervised the whole work. She also contributed to the writing of the original draft and is part of the review&editing.

## Conflicts of interest

There are no conflicts to declare.

## Supplementary Material

RA-015-D5RA06762F-s001

## Data Availability

Please find the repository containing the raw data, evaluated data, evaluation software and DFT calculations under DOI: https://doi.org/10.5281/zenodo.16841978. Supplementary information is available. See DOI: https://doi.org/10.1039/d5ra06762f.
