# Supplementary material for: Charge and size effects in π-ligand activation: an IR spectroscopic study of gold–acetylene complexes
Source: RSC Adv. 2025 Nov 13;15(52):44304–13. doi: 10.1039/d5ra06762f (PMC12612809; doi:10.1039/d5ra06762f)
Supplement: RA-015-D5RA06762F-s001 [file RA-015-D5RA06762F-s001.pdf]

Supplementary Information (SI) for RSC Advances.
This journal is © The Royal Society of Chemistry 2025

Supplementary Information for Charge and Size Effects in π-Ligand Activation: An IR Spectroscopic Study of Gold-Acetylene Complexes

J. Reichegger[a], M. Knabl[a], M. Schmidt[a], A.M. Reider[a], M. Ončák[a], P. Scheier[a], O. V. Lushchikova[a‡]

[a] Institut für Ionenphysik und Angewandte Physik, Universität Innsbruck, Technikerstr. 25, 6020 Innsbruck, Austria
‡ Current address: Department of Chemistry, Faculty of Science, Kyushu University, 744 Motooka, Nishi-ku, 819-0395 Fukuoka, Japan

1 Experimental details

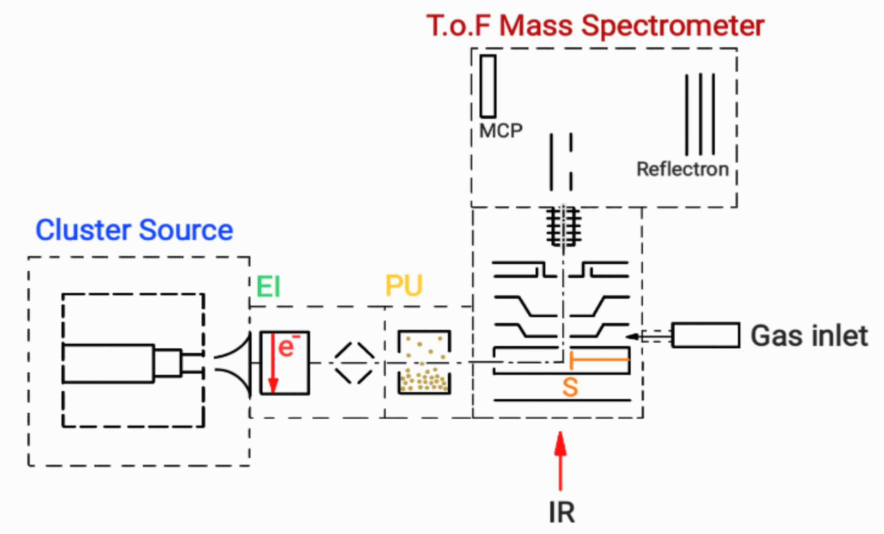

Figure S1: Schematic of the ClusToF apperatus at which the experiment is conducted. A superfluid HND beam is produced in the cluster source and is subsequently multiply ionized by electron impact (EI). The charged HNDs are steered into a resistively heated pick-up oven (PU), where they are doped with atomic gold. Cluster grow around the charge centers is observed. Further pick-up of C_2H_2, which is introduced through a gas inlet, leads to the formation of metal-ligand complexes. These complexes are extracted into a ToF mass spectrometer, after the HNDs collide with a surface (S). IR photodissociation spectrosopy is applied to explore the properties of these metal-ligand complexes.

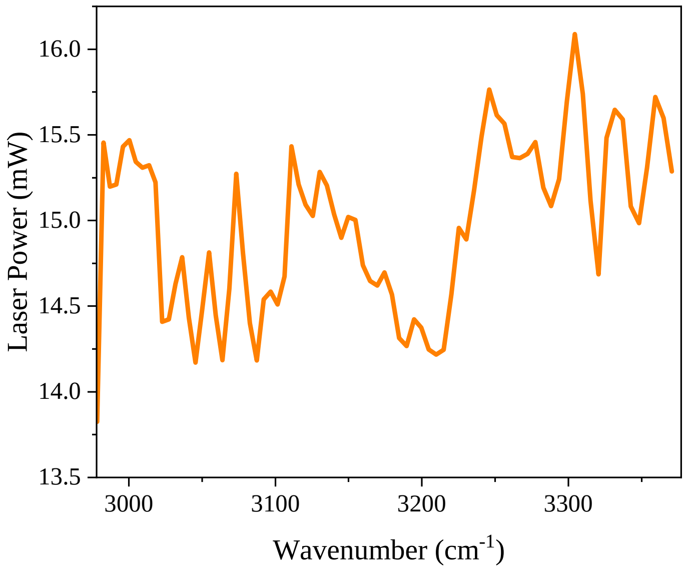

Figure S2: Average laser power in the spectral range of the antisymmetric/symmetric C-H stretch vibration.

Table 1: Overview of the experimental and calculated C-H stretch vibrations.

$Au_n^{+/-}(C_2H_2)_m$	experimental frequencies cm^{-1}	calculated frequencies cm^{-1}	assignment[1]
$Au^+C_2H_2$	3169<br>not visible	3163<br>3247	CH stretch ν_3<br>CH stretch ν_1
$Au^+(C_2H_2)_2$	3160<br>3183<br>3250	none<br>3180<br>3262	acetylene elimination<br>CH stretch ν_3<br>CH stretch ν_1
$Au^+(C_2H_2)_3$	3168<br>3190<br>3250<br>3259	none<br>3185<br>3247<br>3268	acetylene elimination<br>CH stretch ν_3<br>nearly free CH stretch ν_3<br>CH stretch ν_1
$Au_2^+(C_2H_2)$	3189<br>not visible	3184<br>3268	CH stretch ν_3<br>CH stretch ν_1
$Au_2^+(C_2H_2)_2$	3194<br>3259	3195<br>3280	CH stretch ν_3<br>CH stretch ν_1
$Au_2^+(C_2H_2)_3$	3177<br>3196<br>3256	none<br>3198<br>3271	noise<br>CH stretch ν_3<br>CH stretch ν_1

[1] NIST Chemistry WebBook, Acetylene, 1972, https://webbook.nist.gov/cgi/cbook.cgi?ID=C74862&Units=SI&Mask=800#ref-1.

$Au_n^{+/-}(C_2H_2)_m$	experimental frequencies cm^{-1}	calculated frequencies cm^{-1}	assignment
$Au_3^+(C_2H_2)$	3194<br>not visible	3193<br>3278	CH stretch ν_3<br>CH stretch ν_1
$Au_3^+(C_2H_2)_2$	3201<br>not visible	3199<br>3282	CH stretch ν_3<br>CH stretch ν_1
$Au_3^+(C_2H_2)_3$	3207<br>3279	3208<br>3291	CH stretch ν_3<br>CH stretch ν_1
$Au^-C_2H_2$	2923<br>2972<br>3009	3022<br>none<br>none	CH stretch ν_3<br>acetylene elimination<br>acetylene elimination
$Au^-(C_2H_2)_2$	2966<br>3006<br>3057	3051<br>none<br>none	CH stretch ν_3<br>acetylene elimination<br>acetylene elimination
$Au^-(C_2H_2)_3$	3003<br>3033	3072<br>none	CH stretch ν_3<br>acetylene elimination
$Au^-(C_2H_2)_4$	3036	3089	CH stretch ν_3
$Au_2^-C_2H_2$	3135	3167	CH stretch ν_3
$Au_2^-(C_2H_2)_2$	3140	3173	CH stretch ν_3
$Au_2^-(C_2H_2)_3$	3149	3180	CH stretch ν_3
$Au_2^-(C_2H_2)_4$	3159	3187	CH stretch ν_3
$Au_3^-(C_2H_2)$	3160	3185	CH stretch ν_3
$Au_3^-(C_2H_2)_2$	3162	3186	CH stretch ν_3
$Au_3^-(C_2H_2)_3$	3169	3192	CH stretch ν_3
$Au_4^-(C_2H_2)$	3187	3202	CH stretch ν_3
$Au_4^-(C_2H_2)_2$	3193<br>not visible	3181<br>3226	CH stretch ν_3<br>nearly free CH stretch ν_3

2 Structure of calculated cationic clusters as optimized at the ωB97X-D/Def2TZVP

level of theory (Cartesian coordinates, in Å)

Beside the listing of all calculated structures here in the SI, all the xyz-files can also be found in the repository under doi:https://doi.org/10.5281/zenodo.16841978.

Au^+

Au 0.000000000 0.000000000 0.000000000

C_2H_2

C 0.000000000 0.000000000 0.597323000
H 0.000000000 0.000000000 1.661119000
C 0.000000000 0.000000000 -0.597316000
H 0.000000000 0.000000000 -1.661161000

$Au^+C_2H_2$ iso I

C -1.766326000 -0.614822000 -0.000110000
H -2.030733000 -1.656795000 0.000284000
C -1.766383000 0.614816000 0.000084000
Au 0.319716000 -0.000001000 0.000000000
H -2.030543000 1.656874000 -0.000147000

$Au^+(C_2H_2)_2$ iso I

C -2.090312000 0.449799000 0.417532000
H -2.345572000 1.212353000 1.128055000
C -2.090968000 -0.447725000 -0.416120000
Au 0.000005000 -0.000305000 -0.000276000
H -2.348376000 -1.210684000 -1.125440000
C 2.090170000 -0.416522000 0.449168000
H 2.346724000 -1.125922000 1.212334000
C 2.090847000 0.417926000 -0.447699000
H 2.348416000 1.127440000 -1.210425000

$Au^+(C_2H_2)_3$ iso I

Au -0.383052000 0.000953000 0.000017000
C 0.023640000 -2.086748000 0.483448000
C -0.881399000 -2.097447000 -0.341353000
C 0.036667000 2.086344000 -0.483985000
C -0.867955000 2.102786000 0.341132000
H 0.813805000 2.316241000 -1.186388000
H -1.645153000 2.373192000 1.028902000
H -1.660492000 -2.362975000 -1.028885000
H 0.799498000 -2.321994000 1.185505000
C 2.999045000 -0.294930000 -0.524783000
C 3.000353000 0.279357000 0.525357000
H 3.043840000 -0.807581000 -1.458540000
H 3.047485000 0.791677000 1.459179000

$Au^+(C_2H_2)_3$ iso II

Au -0.105187000 0.004487000 -0.000061000
C -1.381383000 1.857631000 -0.000070000
C -0.211396000 2.216997000 0.001770000
C 2.369936000 -0.096562000 -0.602804000
C 2.369589000 -0.103897000 0.603075000
C -0.401347000 -2.192395000 -0.001960000
C -1.535973000 -1.733767000 0.000406000
H -2.595332000 -1.581448000 0.001699000
H 0.436644000 -2.859709000 -0.003161000
H 2.490419000 -0.115549000 1.665488000
H 2.491476000 -0.094845000 -1.665208000
H 0.679925000 2.811228000 0.004172000
H -2.449946000 1.797816000 -0.000650000

$Au^+(C_2H_2)_3$ iso III

Au 0.000000000 0.000983000 0.000000000
C 1.299196000 1.877338000 0.000000000
C 2.080620000 0.940085000 0.000000000
C 0.977573000 -2.065980000 0.000000000
C -0.224502000 -2.274715000 0.000000000
C -1.856865000 1.328791000 0.000000000
C -2.276100000 0.182858000 0.000000000
H 0.864537000 2.854126000 0.000000000
H -1.776684000 2.394919000 0.000000000
H -2.902866000 -0.683244000 0.000000000
H -1.189175000 -2.735483000 0.000000000
H 2.041104000 -2.175411000 0.000000000
H 2.963555000 0.337186000 0.000000000

$Au^+(C_2H_2)_4$ iso I

Au 0.000255000 0.000950000 -0.001585000
C -0.463559000 -2.097996000 -0.394463000
C 0.475237000 -2.095565000 0.391264000
C -0.472242000 2.096973000 0.396716000
C 0.458151000 2.100419000 -0.398887000
C 3.471822000 -0.263752000 -0.531097000
C 3.465749000 0.263876000 0.542932000
C -3.469572000 0.257422000 -0.530652000
C -3.468104000 -0.272537000 0.542223000
H -1.276763000 -2.341557000 -1.049246000
H 1.290080000 -2.333882000 1.045960000
H 1.263525000 2.345627000 -1.062653000
H -1.279801000 2.335272000 1.060329000
H 3.503029000 0.735202000 1.498310000
H 3.516820000 -0.733048000 -1.487143000
H -3.508379000 -0.745871000 1.496490000
H -3.513560000 0.730137000 -1.485057000

$Au^+(C_2H_2)_4$ iso II

Au -0.380747000 0.000514000 -0.040734000
C -0.461510000 2.208870000 -0.222738000
C -0.706771000 1.802223000 -1.350621000
C -0.454393000 -2.206930000 -0.262823000
C -0.702536000 -1.780375000 -1.382446000
H -0.275584000 -2.846578000 0.577244000
H -0.935641000 -1.659679000 -2.419707000
H -0.938268000 1.700090000 -2.390250000
H -1.398630000 -0.038209000 2.883937000
C 3.122281000 0.605245000 -0.502019000
C 3.124138000 -0.590979000 -0.507206000
C 0.771601000 -0.016871000 2.213796000
C -0.398900000 -0.027710000 2.505575000
H 3.158458000 1.670073000 -0.517303000
H -0.286647000 2.836424000 0.627276000
H 3.164309000 -1.655517000 -0.530389000
H 1.827535000 -0.008092000 2.038062000

$Au^+(C_2H_2)_5$ iso I

Au -0.001617000 -0.184280000 0.000198000
C 0.497120000 -0.625824000 -2.084044000
C -0.399005000 0.207443000 -2.112183000
C 0.399145000 0.208456000 2.111385000
C -0.503047000 -0.618324000 2.085095000
C -3.273305000 -1.414559000 -0.358034000
C -3.571132000 -0.379673000 0.163094000
C 3.265436000 -1.433060000 0.361064000
C 3.564695000 -0.401287000 -0.165331000
H 1.277850000 -1.329683000 -2.293465000
H -1.160578000 0.913601000 -2.376259000
H -1.288486000 -1.316287000 2.296595000
H 1.165758000 0.909688000 2.373929000
H -3.878415000 0.531175000 0.622586000
H -3.047217000 -2.343675000 -0.828553000
H 3.872962000 0.506766000 -0.629696000
H 3.038254000 -2.359518000 0.836281000
C 0.517822000 3.262115000 -0.331929000
C -0.479548000 3.266847000 0.328621000
H -1.365129000 3.313095000 0.919058000
H 1.403671000 3.300167000 -0.922538000

Au_2^+

Au 0.000000000 0.000000000 1.334175000
Au 0.000000000 0.000000000 -1.334175000

$Au_2^+C_2H_2$ iso I

C -3.151995000 0.622191000 -0.000288000
C -3.161060000 -0.602733000 0.000165000
Au -1.050857000 -0.002851000 0.000028000
H -3.396984000 1.666496000 0.000277000
H -3.419213000 -1.643863000 -0.000741000
Au 1.616610000 0.001087000 -0.000013000

$Au_2^+(C_2H_2)_2$ iso I

Au -1.347368000 -0.005520000 -0.01066200
Au 1.347312000 -0.008077000 0.009439000
C 3.490720000 0.472703000 0.414954000
C 3.500192000 -0.390508000 -0.449807000
C -3.495315000 0.468322000 -0.406250000
C -3.494934000 -0.399410000 0.454131000
H -3.729281000 1.214395000 -1.138921000
H -3.731016000 -1.136836000 1.194937000
H 3.717731000 1.214301000 1.154395000
H 3.743008000 -1.124306000 -1.191992000

$Au_2^+(C_2H_2)_2$ iso II

C -2.316532000 -1.840480000 -0.235862000
C -1.214332000 -2.145598000 0.205843000
Au -1.185475000 0.031646000 -0.005176000
H -3.320525000 -1.817560000 -0.611789000
H -0.375538000 -2.694010000 0.585478000
Au 1.712525000 -0.062155000 0.004416000
C -0.884350000 2.166492000 -0.507092000
C -1.506388000 2.169873000 0.545322000
H -2.042047000 2.391801000 1.446970000
H -0.369215000 2.428262000 -1.409895000

$Au_2^+(C_2H_2)_3$ iso I

Au 1.396323000 0.000003000 0.000029000
Au -1.330696000 -0.000015000 -0.00002900
C 3.547356000 0.000030000 -0.610502000
C 3.547402000 0.000039000 0.610400000
C -1.362498000 -2.176552000 0.000238000
C -2.560013000 -1.899858000 -0.000310000
H 3.779454000 -0.000198000 1.655785000
H 3.779382000 0.000105000 -1.655893000
H -3.630313000 -1.887715000 -0.000811000
H -0.489658000 -2.797309000 0.000782000
C -1.362686000 2.176788000 -0.000346000
C -2.560095000 1.899797000 0.000518000
H -0.489757000 2.797406000 -0.001127000
H -3.630386000 1.887215000 0.001308000

$Au_2^+(C_2H_2)_4$ iso I

Au 1.414229000 0.000199000 -0.000148000
Au -1.414574000 -0.000127000 -0.00006300
C 2.676808000 1.274806000 1.337107000
C 1.486267000 1.494244000 1.565371000
C 2.679309000 -1.274570000 -1.334563000
C 1.489216000 -1.494428000 -1.564778000
C -1.486600000 1.568638000 -1.490196000
C -2.677233000 1.339802000 -1.271612000
C -2.677260000 -1.340095000 1.271388000
C -1.486666000 -1.569208000 1.489836000
H 0.639513000 1.947850000 2.039158000
H 3.746955000 1.274912000 1.337886000
H 3.749466000 -1.273646000 -1.334480000
H 0.643261000 -1.948565000 -2.039517000
H -0.640027000 -2.044418000 1.942208000
H -3.747436000 -1.341285000 1.271252000
H -3.747400000 1.340735000 -1.272026000
H -0.640078000 2.043568000 -1.943098000

$Au_2^+(C_2H_2)_5$ iso I

Au -1.214862000 -0.511941000 -0.00083300
Au 1.538503000 0.132622000 -0.000220000
C -2.450773000 -0.798815000 1.842566000
C -1.288370000 -0.550081000 2.165425000
C -2.453188000 -0.790306000 -1.843801000
C -1.291308000 -0.539495000 -2.167040000
C 2.113003000 -1.951800000 0.000160000
C 3.199093000 -1.369290000 0.000956000

C 2.322198000 2.231680000 0.000019000
C 1.091542000 2.260784000 -0.000821000
H -0.459173000 -0.381890000 2.821961000
H -3.496325000 -1.026087000 1.836831000
H -3.498625000 -1.018095000 -1.837761000
H -0.463148000 -0.367745000 -2.823969000
H 0.093409000 2.660969000 -0.001569000
H 3.362064000 2.483424000 0.000718000
H 4.240593000 -1.123714000 0.001664000
H 1.444022000 -2.788316000 -0.000352000
C -2.446418000 2.897056000 -0.590825000
C -2.439887000 2.893842000 0.605184000
H -2.453651000 2.908701000 1.670306000
H -2.472114000 2.917545000 -1.655639000

Au_3^+

Au 0.000000000 -1.543918000 0.000000000
Au -1.337749000 0.772875000 0.000000000
Au 1.337749000 0.771044000 0.000000000

$Au_3^+C_2H_2$ iso I

Au 1.377290000 0.000200000 -0.000187000
Au -1.002761000 -1.307553000 0.000130000
Au -1.001986000 1.307680000 -0.000045000
C 3.506317000 -0.002969000 -0.610544000
C 3.506430000 -0.000640000 0.611654000
H 3.746919000 -0.004964000 -1.654900000
H 3.745738000 0.000769000 1.656314000

$Au_3^+(C_2H_2)_2$ iso I

Au -1.409927000 -0.515207000 -0.00708800
Au 1.409888000 -0.515206000 0.007942000
Au 0.000011000 1.711845000 -0.000354000
C -3.107608000 -1.807400000 0.586377000
C -2.943658000 -1.994075000 -0.609833000
H -3.432151000 -1.803123000 1.607100000
H -2.991976000 -2.305677000 -1.633353000
C 3.105912000 -1.807742000 -0.589461000
C 2.945728000 -1.993412000 0.607423000
H 3.426994000 -1.804155000 -1.611275000
H 2.997074000 -2.304414000 1.630974000

$Au_3^+(C_2H_2)_3$ iso I

Au -1.488641000 0.435452000 0.001010000
Au 1.120670000 1.071983000 0.000098000
Au 0.367974000 -1.506771000 0.000888000
C -3.478437000 1.319517000 0.528527000
C -3.638534000 0.751724000 -0.538660000
H -3.560650000 1.865373000 1.445211000
H -3.993599000 0.332159000 -1.456935000
C 2.878861000 2.349320000 0.534567000
C 2.479568000 2.766570000 -0.539500000
H 3.382950000 2.151684000 1.457659000
H 2.305165000 3.278414000 -1.462990000
C 0.596622000 -3.667327000 0.532308000
C 1.161795000 -3.526637000 -0.539275000
H 0.170722000 -4.009784000 1.452472000
H 1.695950000 -3.629282000 -1.460873000

$Au_3^+(C_2H_2)_4$ iso I

Au 0.533210000 1.370866000 -0.227328000
Au -1.699795000 -0.082888000 0.160061000
Au 0.652920000 -1.314585000 -0.207996000
C 1.120005000 3.465354000 -0.747484000
C 1.973417000 3.058296000 0.023430000
H 0.504695000 4.020440000 -1.424287000
H 2.802883000 2.883807000 0.677574000
C -3.914378000 -0.352482000 -0.074703000
C -3.756738000 0.096393000 1.047446000
H -4.272682000 -0.739034000 -1.005870000
H -3.842243000 0.473487000 2.045014000
C 1.751630000 -3.120667000 -0.967967000
C 1.980709000 -3.067206000 0.228044000
H 1.676835000 -3.353049000 -2.009623000
H 2.307618000 -3.192263000 1.239121000
C 3.726619000 0.023666000 1.368008000
C 2.909098000 0.195723000 2.224426000
H 4.466441000 -0.132742000 0.617292000
H 2.193810000 0.346889000 2.999323000

$Au_3^+(C_2H_2)_4$ iso II

Au -0.111687000 -1.539369000 0.001773000
Au 1.386123000 0.799712000 -0.014970000
Au -1.253887000 0.995846000 0.010491000
C -0.256036000 -2.994800000 -1.690762000
C -0.172292000 -1.876237000 -2.190131000
H -0.339187000 -4.053521000 -1.568205000
H -0.114095000 -1.082539000 -2.903032000
C 3.384554000 1.679089000 -0.588000000
C 3.521484000 1.271657000 0.551465000
H 3.477017000 2.098203000 -1.567686000
H 3.850208000 0.994518000 1.530612000
C -3.326079000 1.720926000 -0.524719000
C -3.079316000 2.195445000 0.569423000
H -3.736116000 1.415641000 -1.464004000
H -3.067587000 2.701447000 1.511717000
C -0.107237000 -1.869996000 2.197381000
C -0.194335000 -2.991181000 1.705861000
H -0.045239000 -1.071475000 2.904123000
H -0.272800000 -4.050666000 1.587138000

$Au_3^+(C_2H_2)_5$ iso I

Au 0.001010000 1.396532000 0.000779000
Au 1.292450000 -0.930562000 -0.364003000
Au -1.294078000 -0.928306000 0.364585000
C 0.416067000 3.559799000 0.447834000
C -0.410386000 3.560139000 -0.447857000
H 1.135739000 3.762368000 1.213366000
H -1.129767000 3.762679000 -1.213668000
C 3.414110000 -1.665440000 -0.369660000
C 2.850057000 -2.289268000 -1.251851000
H 4.068442000 -1.217998000 0.349678000
H 2.562172000 -2.932914000 -2.056287000
C -2.856054000 -2.282469000 1.252726000
C -3.416722000 -1.661070000 0.366697000
H -2.570813000 -2.924244000 2.059591000
H -4.068317000 -1.214895000 -0.355896000
C -3.616067000 1.755561000 -1.075064000
C -3.032611000 1.242749000 -1.985035000
H -4.145769000 2.217860000 -0.274266000
H -2.523472000 0.791377000 -2.804936000
C 3.038546000 1.240316000 1.979334000
C 3.619977000 1.752450000 1.067688000
H 4.147864000 2.214124000 0.265334000
H 2.531274000 0.789587000 2.800746000

$Au_3^+(C_2H_2)_5$ iso II

Au -1.112309000 -1.166197000 0.119864000
Au 1.410497000 -0.223691000 -0.544820000
Au -0.497529000 1.546245000 -0.071798000
C -2.555372000 -2.242435000 -1.197051000
C -1.962929000 -1.426206000 -1.898829000
H -3.216826000 -3.017019000 -0.872165000
H -1.653658000 -0.850977000 -2.744499000
C 3.238153000 -1.072633000 -1.569742000
C 3.589652000 -0.785423000 -0.439247000
H 3.147521000 -1.388488000 -2.587266000
H 4.069049000 -0.601023000 0.499646000
C -1.271305000 3.647148000 -0.345558000
C -1.657764000 3.299306000 0.756024000
H -1.041306000 4.142202000 -1.265461000
H -2.087326000 3.197407000 1.730035000
C -0.649900000 -1.372573000 2.288922000
C -1.543654000 -2.185852000 2.077665000
H 0.122674000 -0.785191000 2.743887000
H -2.282102000 -2.949018000 2.198485000
C 2.395644000 0.715317000 2.707397000
C 2.681903000 -0.328385000 3.217309000
H 2.946604000 -1.246434000 3.689719000
H 2.156720000 1.656740000 2.269852000

Au_4^+

Au -2.357211000 -0.000058000 -0.00018700
Au 2.357156000 -0.000165000 -0.000187000
Au 0.000120000 1.360887000 0.000187000
Au -0.000065000 -1.360664000 0.000187000

$Au_4^+C_2H_2$ iso I

Au -2.340618000 -0.224287000 -0.17489200
Au 2.340150000 -0.227803000 -0.175119000
Au -0.001392000 -1.445723000 0.216890000
Au -0.000227000 1.287093000 0.103918000
C 0.623942000 3.402385000 0.165550000
C -0.600809000 3.410697000 0.161411000
H 1.659755000 3.674252000 0.178336000
H -1.633630000 3.694095000 0.166927000

$Au_4^+C_2H_2$ iso II

Au 2.203179000 -0.000096000 -0.000017000
Au -2.602855000 -0.000223000 0.000082000
Au -0.189005000 1.331076000 -0.000078000
Au -0.189811000 -1.330456000 -0.00004200
C 4.361025000 -0.001713000 -0.609805000
C 4.360823000 -0.001529000 0.610422000
H 4.584924000 -0.003093000 -1.657327000
H 4.584837000 -0.001234000 1.657927000

$Au_4^+(C_2H_2)_2$ iso I

Au -0.000316000 1.062357000 -1.478599000
Au 0.000265000 1.096756000 1.455251000
Au -1.459403000 -0.713548000 0.006652000
Au 1.459387000 -0.713618000 0.006412000
C 3.077313000 -2.025498000 0.641931000
C 3.071826000 -2.052081000 -0.586000000
H 3.310782000 -2.185521000 1.675326000
H 3.296650000 -2.257445000 -1.613268000
C -3.072334000 -2.051915000 -0.584813000
C -3.076107000 -2.026711000 0.643168000
H -3.308079000 -2.187770000 1.676738000
H -3.298303000 -2.255821000 -1.612111000

$Au_4^+(C_2H_2)_2$ iso II

Au 2.232327000 -0.241571000 -0.024678000
Au -2.589594000 -0.240179000 -0.05929500
Au -0.150806000 -1.413683000 0.062117000
Au -0.235534000 1.265798000 0.033692000
C -0.790558000 3.384443000 0.029746000
C 0.435333000 3.348021000 0.065444000
H -1.813707000 3.699342000 0.001568000
H 1.470054000 3.619986000 0.098269000
C 4.367301000 0.135390000 0.496652000
C 4.312538000 0.144567000 -0.723239000
H 4.644080000 0.161110000 1.530848000
H 4.496867000 0.186170000 -1.777343000

$Au_4^+(C_2H_2)_3$ iso I

Au -0.000256000 0.000325000 1.883696000
Au 1.531316000 0.394763000 -0.485246000
Au -0.423107000 -1.523054000 -0.48431900
Au -1.107687000 1.127934000 -0.485941000
C -2.504688000 2.549959000 -1.402381000
C -2.650563000 2.694583000 -0.195127000
H -2.579442000 2.628452000 -2.467651000
H -2.948626000 2.994687000 0.788037000
C -1.012390000 -3.641384000 -0.197571000
C -0.956563000 -3.441960000 -1.404578000
H -0.984437000 -3.544897000 -2.470033000
H -1.127414000 -4.050965000 0.784516000
C 3.461337000 0.892651000 -1.402277000
C 3.660156000 0.946636000 -0.195127000
H 3.565956000 0.917999000 -2.467584000
H 4.069284000 1.054283000 0.788029000

$Au_4^+(C_2H_2)_4$ iso I

Au -1.055379000 -0.819532000 -1.08732900
Au -0.818491000 1.055476000 1.086160000
Au 1.052814000 0.818406000 -1.089846000
Au 0.820559000 -1.052814000 1.088599000
C -2.832997000 -2.209194000 -1.031794000
C -2.559533000 -1.988858000 -2.203002000
H -3.234669000 -2.528509000 -0.093839000
H -2.548016000 -1.973526000 -3.273253000
C 2.206586000 -2.833612000 1.041508000
C 1.987888000 -2.552759000 2.211263000
H 2.523543000 -3.241901000 0.105614000
H 1.974283000 -2.534480000 3.281452000
C -1.980537000 2.552666000 2.216268000
C -2.207829000 2.832810000 1.047851000

H -1.959401000 2.535990000 3.286372000
H -2.533861000 3.240549000 0.114813000
C 2.838676000 2.196975000 -1.042769000
C 2.553395000 1.984614000 -2.212703000
H 3.251016000 2.509315000 -0.107091000
H 2.532427000 1.975377000 -3.282893000

$Au_4^+(C_2H_2)_4$ iso II

Au -2.169411000 0.000491000 0.492862000
Au 2.169393000 -0.000257000 0.492872000
Au -0.000253000 -1.336862000 -0.56572000
Au 0.000070000 1.336624000 -0.566002000
C 0.614884000 3.382499000 -1.141568000
C -0.611756000 3.383632000 -1.141707000
H 1.642287000 3.669103000 -1.222865000
H -1.638768000 3.671576000 -1.223078000
C -0.613837000 -3.383573000 -1.140785000
C 0.612770000 -3.383805000 -1.140890000
H 1.640039000 -3.670880000 -1.222142000
H -1.641174000 -3.670443000 -1.221831000
C 4.353594000 -0.000735000 1.013043000
C 3.773295000 0.001375000 2.085294000v
H 5.057995000 -0.002396000 0.207957000
H 3.464281000 0.003097000 3.109089000
C -3.773235000 0.001154000 2.085198000
C -4.353458000 -0.000604000 1.012892000
H -3.464256000 0.002664000 3.109005000
H -5.058110000 -0.002107000 0.208009000

$Au_4^+(C_2H_2)_5$ iso I

Au 2.096646000 -0.014873000 0.422155000
Au -2.096631000 -0.013736000 0.422243000
Au 0.000232000 1.372560000 -0.716700000
Au -0.000169000 -1.297183000 -0.82951700
C -0.615004000 -3.298277000 -1.529589000
C 0.612414000 -3.298730000 -1.530001000
H -1.641201000 -3.582795000 -1.631866000
H 1.638323000 -3.584043000 -1.632980000
C 0.615340000 3.423355000 -1.251369000
C -0.612328000 3.424235000 -1.250999000
H -1.637648000 3.719060000 -1.330801000
H 1.641011000 3.716787000 -1.331796000
C -4.211979000 -0.041291000 1.170284000
C -3.514804000 -0.112123000 2.168660000
H -5.006662000 0.012195000 0.456357000
H -3.072033000 -0.178146000 3.140432000
C 3.514120000 -0.110430000 2.169308000
C 4.211779000 -0.041714000 1.171142000
H 3.070870000 -0.174767000 3.140983000
H 5.006995000 0.010248000 0.457698000
C -0.000611000 0.354946000 3.298067000
C 0.000189000 -0.826889000 3.483179000
H 0.000887000 -1.879020000 3.650855000
H -0.001288000 1.407357000 3.132689000

$Au_4^+(C_2H_2)_5$ iso II

Au 0.787192000 -1.211859000 -0.772574000
Au -1.746383000 -0.517748000 0.410078000
Au -0.128305000 1.265776000 -1.142878000
Au 0.451049000 0.460742000 1.567733000
C 1.454786000 -3.345801000 -0.421988000
C 1.995612000 -2.940558000 -1.440047000
H 1.086027000 -3.886683000 0.423012000
H 2.586611000 -2.839327000 -2.326472000
C 2.402101000 1.148474000 2.492901000
C 1.540250000 1.201200000 3.356967000
H 3.265370000 1.146059000 1.858327000
H 1.002640000 1.341904000 4.271407000
C -3.816108000 -1.257744000 0.602428000
C -3.575195000 -1.364405000 -0.592071000
H -4.285599000 -1.274409000 1.564204000
H -3.579239000 -1.539020000 -1.646861000
C -0.790428000 3.401965000 -1.370223000
C -0.313652000 3.035081000 -2.436295000
H -1.239799000 3.923940000 -0.552368000
H 0.024407000 2.998475000 -3.451348000
C 3.858111000 0.511025000 -0.741417000
C 4.429240000 -0.353571000 -0.143939000
H 4.949594000 -1.123151000 0.377579000
H 3.361058000 1.282326000 -1.281699000

$Au_4^+(C_2H_2)_5$ iso III

Au -2.173336000 0.000430000 -0.546980000
Au 2.008453000 -0.000202000 -0.831043000
Au -0.003540000 1.336926000 0.501973000
Au -0.003737000 -1.337419000 0.500667000
C 0.648366000 -3.352725000 1.127859000
C -0.577935000 -3.365342000 1.164671000
H 1.679831000 -3.627721000 1.193909000
H -1.598034000 -3.664141000 1.283580000
C -0.575523000 3.365392000 1.166194000
C 0.650802000 3.350939000 1.131309000
H 1.682640000 3.624107000 1.199103000
H -1.595369000 3.665629000 1.283650000
C 4.095929000 -0.003574000 -1.656298000
C 3.374278000 0.007769000 -2.638753000
H 4.911814000 -0.013236000 -0.964923000
H 2.922105000 0.017696000 -3.607612000
C -3.781228000 0.001634000 -2.136250000
C -4.357743000 0.000670000 -1.061930000
H -3.476466000 0.002554000 -3.161148000
H -5.062059000 -0.000079000 -0.256948000
C 0.677589000 -0.001223000 3.797842000
C 1.788780000 -0.000546000 3.356163000
H 2.783033000 0.000043000 2.977694000
H -0.306772000 -0.001824000 4.203144000

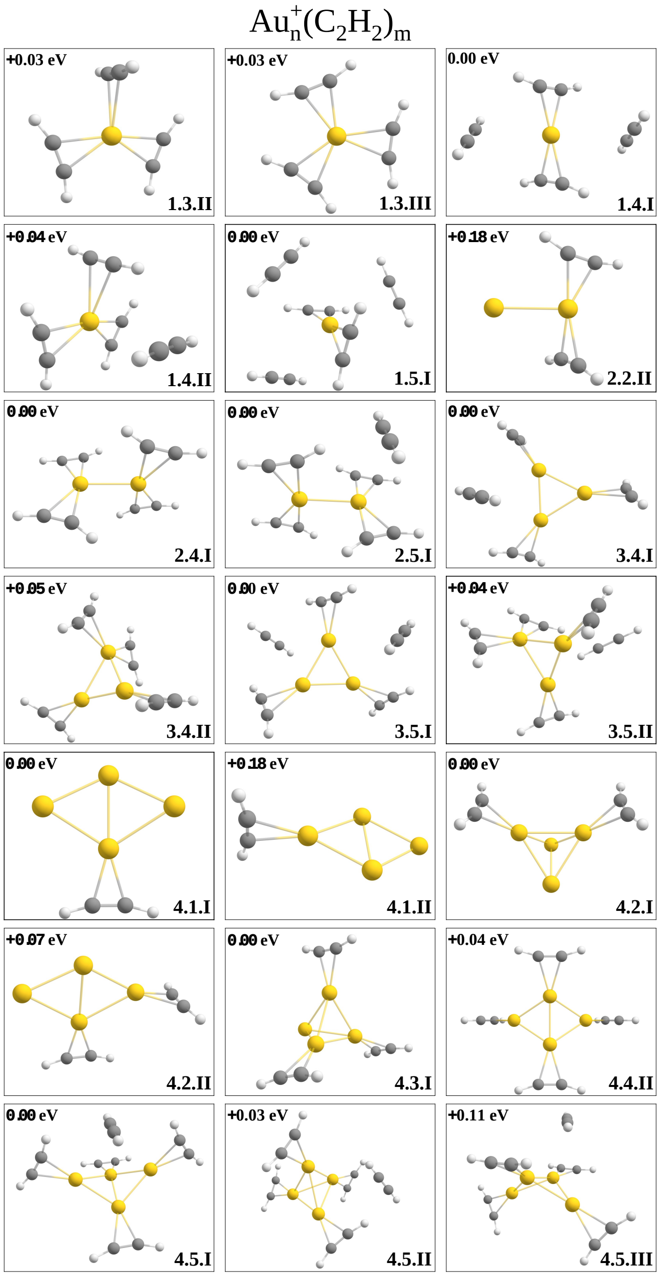

Figure S3: Further calculated structures of $Au_n^+(C_2H_2)_m$. They were not considered for the experimental spectra, due to them either not being the ground state or their IR spectra did not match the experiment or due to missing experimental spectra to compare to.

3 Structure of calculated anionic clusters as optimized at the ωB97X-D/Def2TZVP

level of theory (Cartesian coordinates, in Å)

Au^-

Au 0.000000000 0.632157000 0.000000000

$Au^-C_2H_2$ iso I

Au 0.000000000 0.656493000 0.000000000
H -0.000135000 -2.021191000 0.000000000
C -0.000050000 -3.106794000 0.000000000
C 0.000051000 -4.305463000 0.000000000
H 0.000128000 -5.368236000 0.000000000

$Au^-(C_2H_2)_2$ iso I

Au 0.000000000 0.000000000 0.000787000
H 0.000000000 0.000000000 -2.700920000
H 0.000000000 0.000000000 2.696339000
C 0.000000000 0.000000000 -3.783762000
C 0.000000000 0.000000000 -4.981934000
C 0.000000000 0.000000000 3.779307000
C 0.000000000 0.000000000 4.977515000
H 0.000000000 0.000000000 6.040235000
H 0.000000000 0.000000000 -6.044589000

$Au^-(C_2H_2)_3$ iso I

Au 0.000000000 0.000000000 -0.058120000
H 0.000000000 2.263045000 1.455157000
H 0.000000000 -2.263045000 1.455157000
C 0.000000000 3.160118000 2.058975000
C 0.000000000 4.153637000 2.728211000
C 0.000000000 -3.160118000 2.058975000
C 0.000000000 -4.153637000 2.728211000
H 0.000000000 -5.034772000 3.322503000
H 0.000000000 5.034772000 3.322503000
H 0.000000000 0.000000000 -2.778943000
H 0.000000000 0.000000000 -6.120948000
C 0.000000000 0.000000000 -5.058125000
C 0.000000000 0.000000000 -3.860234000

$Au^-(C_2H_2)_4$ iso I

Au 0.008530000 0.017255000 -0.011010000
C -2.496691000 2.833007000 -0.618468000
C -3.281511000 3.717012000 -0.810563000
C 3.593034000 1.296580000 -0.310635000
C 4.717520000 1.697966000 -0.404114000
H -3.978125000 4.501580000 -0.980348000
H -1.787927000 2.036499000 -0.446181000
H 5.715329000 2.054720000 -0.486291000
H 2.579008000 0.934544000 -0.226272000
C -0.774422000 -3.689493000 -3.297097000
C -0.587708000 -2.804077000 -2.512559000
H -0.939975000 -4.475136000 -3.993518000
H -0.419513000 -2.005922000 -1.804630000
C -0.546324000 -1.400858000 3.489566000
C -0.720368000 -1.845118000 4.588057000
H -0.389241000 -1.000111000 2.498984000
H -0.874568000 -2.239470000 5.562920000

Au_2^-

Au 0.000000000 0.000000000 1.348898000
Au 0.000000000 0.000000000 -1.348898000

$Au_2^-C_2H_2$ iso I

Au -1.343414000 -0.368760000 0.000005000
Au 1.350926000 -0.343300000 0.000005000
C -0.035455000 3.420362000 -0.000243000
C -0.049296000 4.617497000 0.000113000
H -0.061692000 5.680112000 0.000616000
H -0.023225000 2.345466000 -0.000591000

$Au_2^-(C_2H_2)_2$ iso I

Au -0.000326000 -1.345203000 -0.08498200
Au 0.000367000 1.347470000 -0.083991000
C 3.743295000 -0.003905000 0.401493000

C 4.930842000 -0.009265000 0.552166000
C -3.743496000 -0.006541000 0.401694000
C -4.931095000 -0.005859000 0.552068000
H 5.985052000 -0.014199000 0.686364000
H 2.677416000 0.000899000 0.265944000
H -2.677577000 -0.007201000 0.266438000
H -5.985410000 -0.005194000 0.685564000

$Au_2^-(C_2H_2)_3$ iso I

Au 0.000000000 -1.345282000 -0.044904000
Au 0.000000000 1.345282000 -0.044904000
C 0.000000000 0.000000000 3.739514000
C 0.000000000 0.000000000 4.936416000
H 0.000000000 0.000000000 2.665660000
H 0.000000000 0.000000000 5.999174000
H 2.450157000 0.000539000 -1.201226000
H -2.450157000 -0.000539000 -1.201226000
C 3.421147000 -0.000067000 -1.659922000
C 4.503375000 -0.000781000 -2.171210000
C -3.421147000 0.000067000 -1.659922000
C -4.503375000 0.000781000 -2.171210000
H 5.464498000 -0.001326000 -2.624773000
H -5.464498000 0.001326000 -2.624773000

$Au_2^-(C_2H_2)_4$ iso I

Au -0.001230000 0.001186000 -1.344055000
Au -0.000916000 0.000828000 1.343966000
C -2.812309000 2.549830000 0.000311000
C -3.698726000 3.353765000 0.000419000
C 2.809851000 -2.547724000 -0.000346000
C 3.695980000 -3.351974000 -0.000472000
H -4.485704000 4.068017000 0.000692000
H -2.017252000 1.829086000 0.000140000
H 2.014849000 -1.826921000 -0.000213000
H 4.483132000 -4.066032000 -0.000591000
H 1.832543000 2.011807000 -0.007009000
H -1.823597000 -2.019917000 0.007129000
C 2.555445000 2.804924000 -0.002727000
C 3.362208000 3.688750000 0.002071000
C -2.542930000 -2.816275000 0.003088000
C -3.345366000 -3.704043000 -0.001380000
H 4.078501000 4.473854000 0.006590000
H -4.057905000 -4.492559000 -0.005516000

Au_3^-

Au -0.000033000 0.000156000 -0.000337000
Au 0.241143000 -0.324237000 2.579123000
Au -0.241110000 0.324081000 -2.578787000

$Au_3^-C_2H_2$ iso I

Au 0.000000000 0.267200000 0.000000000
Au 2.497799000 -0.486877000 0.000000000
Au -2.501068000 0.990617000 0.000000000
C 0.181161000 -3.775293000 0.000000000
C -0.144699000 -4.926755000 0.000000000
H 0.481057000 -2.744767000 0.000000000
H -0.441539000 -5.947225000 0.000000000

$Au_3^-C_2H_2$ iso II

Au -0.610111000 1.500003000 0.000172000
Au -0.675215000 -1.476844000 0.000181000
Au 1.754647000 -0.032989000 -0.000167000
C -2.503223000 0.715146000 -0.000879000
C -2.531577000 -0.610044000 -0.000834000
H -3.461520000 -1.176143000 -0.002746000
H -3.406053000 1.322095000 -0.001679000

$Au_3^-C_2H_2$ iso III

Au 0.000000000 0.928105000 0.000000000
Au 2.073437000 -0.732172000 0.000000000
Au -2.073376000 -0.732703000 0.000000000
C 0.623992000 2.966465000 0.000000000
C -0.624702000 2.966318000 0.000000000
H 1.600473000 3.403983000 0.000000000
H -1.601019000 3.404163000 0.000000000

$Au_3^-(C_2H_2)_2$ iso I

Au 0.000000000 0.001463000 0.000000000
Au 2.520729000 0.651389000 0.000000000
Au -2.519580000 -0.651333000 0.000000000
C 2.317196000 -3.353817000 0.000000000
C 2.625154000 -4.510258000 0.000000000

H 2.048914000 -2.314602000 0.000000000
H 2.890524000 -5.539472000 0.000000000
H -2.896419000 5.531021000 0.000000000
C -2.631524000 4.501711000 0.000000000
C -2.323769000 3.345247000 0.000000000
H -2.056191000 2.305785000 0.000000000

$Au_3^-(C_2H_2)_2$ iso II

Au 0.000000000 0.145964000 0.000000000
Au 0.033128000 -2.461866000 0.000000000
Au -0.038761000 2.743033000 0.000000000
C -3.805982000 -1.235655000 0.000000000
C -5.001693000 -1.280784000 0.000000000
H -2.733790000 -1.202434000 0.000000000
H -6.063989000 -1.313703000 0.000000000
H 6.096708000 -1.149693000 0.000000000
C 5.033909000 -1.153926000 0.000000000
C 3.837350000 -1.150188000 0.000000000
H 2.764574000 -1.154184000 0.000000000

$Au_3^-(C_2H_2)_2$ iso III

Au -0.203144000 -0.281216000 0.000212000
Au 2.376690000 -0.096414000 0.000062000
Au -2.826941000 -0.385022000 -0.00017800
C 5.149752000 0.834475000 -0.001246000
C 5.350690000 -0.346008000 -0.000047000
H 4.961693000 1.881557000 -0.002298000
H 5.500323000 -1.398862000 0.001056000
H -1.559702000 2.388110000 -0.000890000
C -1.557640000 3.462086000 -0.000254000
C -1.563129000 4.658951000 0.000453000
H -1.562130000 5.721666000 0.001125000

$Au_3^-(C_2H_2)_3$ iso I

Au -0.061019000 0.003081000 -0.019854000
Au -2.598120000 0.038784000 -0.573209000
Au 2.483831000 -0.029509000 0.520857000
C -2.251529000 -0.217338000 3.410102000
C -2.495269000 -0.293955000 4.579037000
H -2.041055000 -0.148398000 2.360278000
H -2.702465000 -0.362770000 5.619193000
H 1.862474000 5.480810000 -2.281494000
C 1.753293000 4.520443000 -1.839451000
C 1.621697000 3.439480000 -1.343614000
H 1.511038000 2.470385000 -0.897613000
C 1.737535000 -4.306381000 -2.298013000
C 1.612695000 -3.281668000 -1.693020000
H 1.507481000 -2.363123000 -1.149307000
H 1.841269000 -5.216474000 -2.837075000

$Au_3^-(C_2H_2)_3$ iso II

Au 0.202321000 0.001314000 -0.002836000
Au 2.793672000 -0.000460000 0.003029000
Au -2.403829000 0.008178000 -0.010904000
C -1.074973000 3.684171000 -1.051461000
C -1.122819000 4.834515000 -1.376911000
H -1.039129000 2.653078000 -0.759522000
H -1.159365000 5.856627000 -1.665958000
H -1.149597000 -4.464091000 -4.131855000
C -1.125268000 -3.681176000 -3.413482000
C -1.090583000 -2.800940000 -2.603873000
H -1.066728000 -2.011896000 -1.878152000
C -1.153857000 -1.214532000 4.863211000
C -1.115285000 -0.923898000 3.703244000
H -1.087895000 -0.663175000 2.663497000
H -1.181495000 -1.472901000 5.893782000

$Au_3^-(C_2H_2)_3$ iso III

Au 0.282409000 -0.039725000 -0.000860000
Au 2.845074000 -0.706442000 -0.000321000
Au -2.222442000 0.548001000 -0.000841000
C 2.576972000 3.252012000 0.004586000
C 2.777896000 4.432091000 0.006558000
H 2.406693000 2.190437000 0.002848000
H 2.947534000 5.481179000 0.008370000
H -4.454201000 -4.460535000 0.007943000
C -3.627772000 -3.791774000 0.006546000
C -2.682787000 -3.056362000 0.005023000
H -1.859926000 -2.366357000 0.003595000
C -4.584378000 0.096799000 0.001012000
C -4.647418000 1.303309000 -0.000919000
H -4.747422000 2.362546000 -0.002552000

H -4.665954000 -0.968621000 0.002694000

$Au_3^-(C_2H_2)_4$ iso I

Au 0.000714000 0.004688000 -0.002346000
Au -2.500825000 0.005799000 -0.697650000
Au 2.502198000 0.001158000 0.693484000
C 1.935051000 -2.971929000 -1.969721000
C 2.118987000 -3.896001000 -2.707021000
H 1.777087000 -2.143980000 -1.307012000
H 2.273904000 -4.716920000 -3.364084000
H -2.299458000 -4.686830000 3.388575000
C -2.139103000 -3.870313000 2.727345000
C -1.949152000 -2.951235000 1.985349000
H -1.785734000 -2.127807000 1.318364000
C 2.146207000 3.854532000 -2.759321000
C 1.959088000 2.940698000 -2.010159000
H 1.798214000 2.121982000 -1.336742000
H 2.303880000 4.666314000 -3.426996000
H -2.298318000 4.642906000 3.454528000
C -2.140788000 3.835745000 2.781247000
C -1.953863000 2.927076000 2.025786000
H -1.793043000 2.112998000 1.346759000

$Au_3^-(C_2H_2)_4$ iso II

Au 0.000320000 -0.000817000 -0.469460000
Au -2.407923000 -0.979491000 -0.39780300
Au 2.408847000 0.976876000 -0.400841000
C 0.123271000 -1.748645000 2.717157000
C 1.116272000 -2.192668000 3.214554000
H -0.741399000 -1.347773000 2.233153000
H 2.015550000 -2.585358000 3.623018000
H -2.569736000 1.917487000 0.359415000
C -3.204529000 2.765336000 0.526455000
C -3.926017000 3.701848000 0.712669000
H -4.561476000 4.538622000 0.872936000
C 3.914985000 -3.706252000 0.722938000
C 3.195796000 -2.768356000 0.534921000
H 2.562862000 -1.919484000 0.366423000
H 4.547958000 -4.544528000 0.885108000
H -2.014368000 2.605402000 3.609544000
C -1.113941000 2.214946000 3.201439000
C -0.119762000 1.772673000 2.704795000
H 0.745878000 1.373468000 2.221079000

Au_4^- iso I

Au 2.330302000 0.001820000 0.000000000
Au 0.000000000 1.436453000 0.000000000
Au 0.000023000 -1.438215000 0.000000000
Au -2.330325000 -0.000058000 0.000000000

Au_4^- iso II

Au 0.556767000 -3.891662000 0.000000000
Au -0.000212000 -1.329859000 0.000000000
Au -0.556555000 3.891684000 0.000000000
Au 0.000000000 1.329838000 0.000000000

Au_4^- iso III

Au -1.386140000 -1.830775000 0.000000000
Au 0.000000000 0.536167000 0.000000000
Au 0.001681000 3.126371000 0.000000000
Au 1.384460000 -1.831763000 0.000000000

$Au_4^-C_2H_2$ iso I

Au -0.029804000 -3.817558000 0.000000000
Au -0.539536000 -1.248790000 0.000000000
Au -0.136507000 3.954193000 0.000000000
Au 0.000000000 1.342777000 0.000000000
C 3.385729000 -1.318915000 0.000000000
C 4.581551000 -1.284582000 0.000000000
H 2.314610000 -1.351041000 0.000000000
H 5.643679000 -1.247090000 0.000000000

$Au_4^-C_2H_2$ iso II

Au 0.051081000 -2.200032000 0.000000000
Au 0.000000000 0.549305000 0.000000000
Au 1.521365000 2.642116000 0.000000000
Au -2.356180000 -0.817451000 0.000000000
C 4.958944000 -1.247739000 0.000000000
C 3.887362000 -0.715402000 0.000000000
H 5.907433000 -1.727053000 0.000000000
H 2.929748000 -0.235200000 0.000000000

$Au_4^-C_2H_2$ iso III

Au -2.486373000 0.093457000 0.000000000
Au 0.000000000 1.247730000 0.000000000
Au -0.339937000 -1.599155000 0.000000000
Au 2.147189000 -0.438005000 0.000000000
C 3.439031000 3.477260000 0.000000000
C 4.226224000 4.378472000 0.000000000
H 2.734326000 2.667804000 0.000000000
H 4.924670000 5.179715000 0.000000000

$Au_4^-(C_2H_2)_2$ iso I

Au -3.974712000 0.205179000 -0.000046000
Au -1.486792000 -0.621346000 0.000403000
Au 3.713595000 -0.629392000 -0.000278000
Au 1.131323000 -0.233735000 -0.000047000
C -0.877237000 3.061328000 -0.001897000
C 0.142361000 3.687194000 -0.002594000
H -1.763226000 2.464507000 -0.001284000
H 1.075762000 4.199909000 -0.003170000
H 3.391196000 2.242099000 0.001481000
C 3.679607000 3.275836000 0.001854000
C 4.006758000 4.426985000 0.002268000
H 4.297595000 5.449607000 0.002669000

$Au_4^-(C_2H_2)_2$ iso II

Au -3.858892000 -0.195449000 0.000314000
Au -1.254505000 -0.410778000 -0.00044700
Au 3.858340000 0.197207000 0.000093000
Au 1.253673000 0.409145000 0.000163000
C -1.737853000 3.480734000 -0.000437000
C -1.766508000 4.676696000 -0.000550000
H -1.711208000 2.409520000 -0.000333000
H -1.785152000 5.739362000 -0.000645000
H 1.792952000 -5.740070000 -0.000402000
C 1.774319000 -4.677403000 -0.000279000
C 1.745653000 -3.481442000 -0.000125000
H 1.719049000 -2.410234000 0.000005000

$Au_4^-(C_2H_2)_2$ iso III

Au 1.957063000 1.390131000 0.008826000
Au -0.391870000 0.000635000 -0.000823000
Au -2.975084000 -0.000558000 -0.00107100
Au 1.956578000 -1.390181000 -0.008984000
C -1.613186000 -5.069394000 0.024188000
C -1.469672000 -3.881645000 0.017315000
H -1.344635000 -2.817898000 0.011234000
H -1.733878000 -6.125266000 0.030225000
H -1.348189000 2.817618000 -0.004813000
C -1.472620000 3.881447000 -0.007587000
C -1.615501000 5.069289000 -0.010744000
H -1.735682000 6.125232000 -0.013556000

$Au_4^-(C_2H_2)_2$ iso IV

Au 3.713016000 -0.125451000 -0.000815000
Au 1.184415000 -0.802682000 0.000641000
Au -3.968688000 -0.005010000 -0.00028600
Au -1.361847000 -0.099448000 -0.00027500
C 1.222990000 2.433241000 2.252851000
C 1.221272000 3.390294000 2.970297000
H 1.225910000 1.575824000 1.611274000
H 1.213365000 4.243203000 3.604305000
H 1.212818000 4.247918000 -3.599012000
C 1.221351000 3.394473000 -2.965734000
C 1.223706000 2.436810000 -2.249108000
H 1.227231000 1.578843000 -1.608281000

$Au_4^-(C_2H_2)_2$ iso V

Au 2.333942000 -0.364972000 -0.000324000
Au 0.000128000 1.060506000 0.000123000
Au -0.000380000 -1.796530000 -0.00013000
Au -2.334257000 -0.364132000 0.000404000
C -3.182948000 3.652302000 -0.001402000
C -3.888295000 4.618774000 -0.001703000
H -2.553154000 2.783878000 -0.001071000
H -4.513156000 5.478646000 -0.001994000
H 4.521908000 5.472240000 0.001030000
C 3.893460000 4.614986000 0.001106000
C 3.184180000 3.651395000 0.001175000
H 2.550846000 2.785575000 0.001186000

$Au_4^-(C_2H_2)_3$ iso I

Au 4.017027000 0.234460000 0.000032000
Au 1.500121000 -0.486278000 -0.000042000
Au -3.651475000 -0.380299000 -0.00001000
Au -1.080672000 0.067379000 -0.000106000
C 1.018801000 3.207808000 0.000143000
C -0.006959000 3.823695000 0.000137000
H 1.909373000 2.618229000 0.000083000
H -0.944466000 4.329324000 0.000245000
H -1.167528000 -6.107617000 0.000615000
C -1.201305000 -5.045305000 0.000436000
C -1.233319000 -3.849425000 0.000237000
H -1.262106000 -2.778238000 0.000165000
C -3.585275000 3.540384000 0.000165000
C -3.845240000 4.708489000 0.000274000
H -3.354294000 2.492438000 0.000168000
H -4.076234000 5.746337000 0.000364000

$Au_4^-(C_2H_2)_3$ iso II

Au -3.773518000 0.270405000 -0.000239000
Au -1.186492000 0.636538000 -0.000670000
Au 3.882499000 -0.258302000 0.000176000
Au 1.274917000 -0.298792000 -0.000963000
C -1.549829000 -2.573209000 2.241626000
C -1.561021000 -3.540014000 2.945737000
H -1.538984000 -1.707398000 1.611580000
H -1.564139000 -4.401476000 3.568178000
H -1.565830000 -4.409814000 -3.558142000
C -1.561974000 -3.547286000 -2.937182000
C -1.550028000 -2.579267000 -2.234752000
H -1.538461000 -1.712293000 -1.606321000
H 1.929911000 2.477398000 0.000947000
C 1.974061000 3.547997000 0.001539000
C 2.020082000 4.743408000 0.002199000
H 2.054608000 5.805710000 0.002759000

$Au_4^-(C_2H_2)_3$ iso III

Au -1.800288000 1.220745000 -0.200682000
Au -0.220927000 -0.481165000 1.228147000
Au 0.606731000 0.617063000 -1.342719000
Au 2.215142000 -1.046459000 0.109067000
C 1.696995000 2.981465000 1.655851000
C 2.206649000 3.923933000 2.188104000
H 1.240867000 2.138422000 1.178761000
H 2.659556000 4.761078000 2.660799000
H -6.255553000 -2.761229000 1.069918000
C -5.302450000 -2.328981000 0.884091000
C -4.229638000 -1.839552000 0.680298000
H -3.272329000 -1.394701000 0.496813000
H -3.165270000 -3.898770000 -1.667552000
C -2.223715000 -3.410600000 -1.597036000
C -1.181051000 -2.833983000 -1.490696000
H -0.260028000 -2.303004000 -1.373576000

$Au_4^-(C_2H_2)_4$ iso I

Au -3.806549000 -0.340174000 0.000050000
Au -1.205556000 -0.526602000 0.000391000
Au 3.805728000 0.345062000 -0.000385000
Au 1.204395000 0.522179000 -0.000100000
C -1.722410000 2.671680000 -2.204759000
C -1.724210000 3.654043000 -2.887015000
H -1.717970000 1.792158000 -1.594056000
H -1.718968000 4.529054000 -3.490283000
H -1.719045000 4.532989000 3.488084000
C -1.724400000 3.657876000 2.884969000
C -1.722623000 2.675362000 2.202942000
H -1.718273000 1.795693000 1.592496000
H 1.721193000 -1.796605000 -1.593018000
C 1.726414000 -2.676045000 -2.203813000
C 1.729168000 -3.658317000 -2.886184000
H 1.724914000 -4.533204000 -3.489635000
C 1.728815000 -2.673758000 2.206040000
C 1.731578000 -3.656092000 2.888295000
H 1.727219000 -4.530947000 3.491774000
H 1.723551000 -1.794348000 1.595213000

$Au_4^-(C_2H_2)_4$ iso II

Au 3.922004000 -0.583195000 0.014571000
Au 1.452419000 0.290402000 0.034862000
Au -3.704877000 0.387241000 0.041866000
Au -1.149886000 -0.148638000 0.034145000

C 0.733161000 -3.394376000 -0.098511000
C -0.295376000 -4.004288000 -0.138690000
H 1.628093000 -2.812403000 -0.062597000
H -1.235286000 -4.503747000 -0.173512000
H -1.865276000 6.052746000 -0.177575000
C -1.722661000 4.999827000 -0.142108000
C -1.572046000 3.813300000 -0.101305000
H -1.444621000 2.749470000 -0.064694000
C -3.825274000 -3.526989000 -0.187533000
C -4.178188000 -4.668337000 -0.256479000
H -3.512225000 -2.502724000 -0.125846000
H -4.492415000 -5.682202000 -0.317966000
H 3.837159000 2.699070000 -0.227459000
C 2.996914000 3.354257000 -0.241062000
C 2.017210000 4.040444000 -0.251676000
H 1.109020000 4.597776000 -0.256232000

$Au_4^-(C_2H_2)_4$ iso III

Au 0.367669000 2.973176000 0.000000000
Au 0.000000000 0.420222000 0.000000000
Au 0.908488000 -2.148127000 0.000000000
Au -1.824270000 -1.619248000 0.000000000
C 3.899859000 0.722307000 0.000000000
C 4.935625000 1.321049000 0.000000000
C -3.684177000 2.035251000 0.000000000
C -4.868717000 2.202732000 0.000000000
C 3.848271000 4.589980000 0.000000000
C 4.514935000 5.583498000 0.000000000
C -1.152435000 -5.525648000 0.000000000
C -1.303586000 -6.712280000 0.000000000
H -1.017583000 -4.462590000 0.000000000
H -1.436834000 -7.766817000 0.000000000
H 2.966308000 0.199049000 0.000000000
H 5.843021000 1.874842000 0.000000000
H -2.623781000 1.886808000 0.000000000
H -5.922498000 2.340987000 0.000000000
H 3.250724000 3.701446000 0.000000000
H 5.102948000 6.469130000 0.000000000

$Au_4^-(C_2H_2)_4$ iso IV

Au 2.574186000 0.042309000 0.001432000
Au 0.265223000 -0.953434000 1.086458000
Au 0.235971000 0.962138000 -1.087450000
Au -2.059599000 -0.032965000 -0.00143200
C -3.231202000 4.981346000 -0.397496000
C -2.579882000 3.983731000 -0.286340000
C 0.311529000 -2.792372000 -2.130917000
C -0.266098000 -3.818941000 -2.338904000
C 0.212045000 2.799741000 2.131798000
C -0.404075000 3.801953000 2.348015000
C -2.441215000 -4.068789000 0.290159000
C -3.055676000 -5.090234000 0.394765000
H -2.000243000 3.088278000 -0.187481000
H -3.810787000 5.866239000 -0.501729000
H 0.814326000 -1.872754000 -1.919894000
H -0.801326000 -4.724128000 -2.493967000
H 0.748912000 1.901301000 1.913484000
H -0.972744000 4.685091000 2.510930000
H -1.894837000 -3.152003000 0.197491000
H -3.602561000 -5.996368000 0.493102000

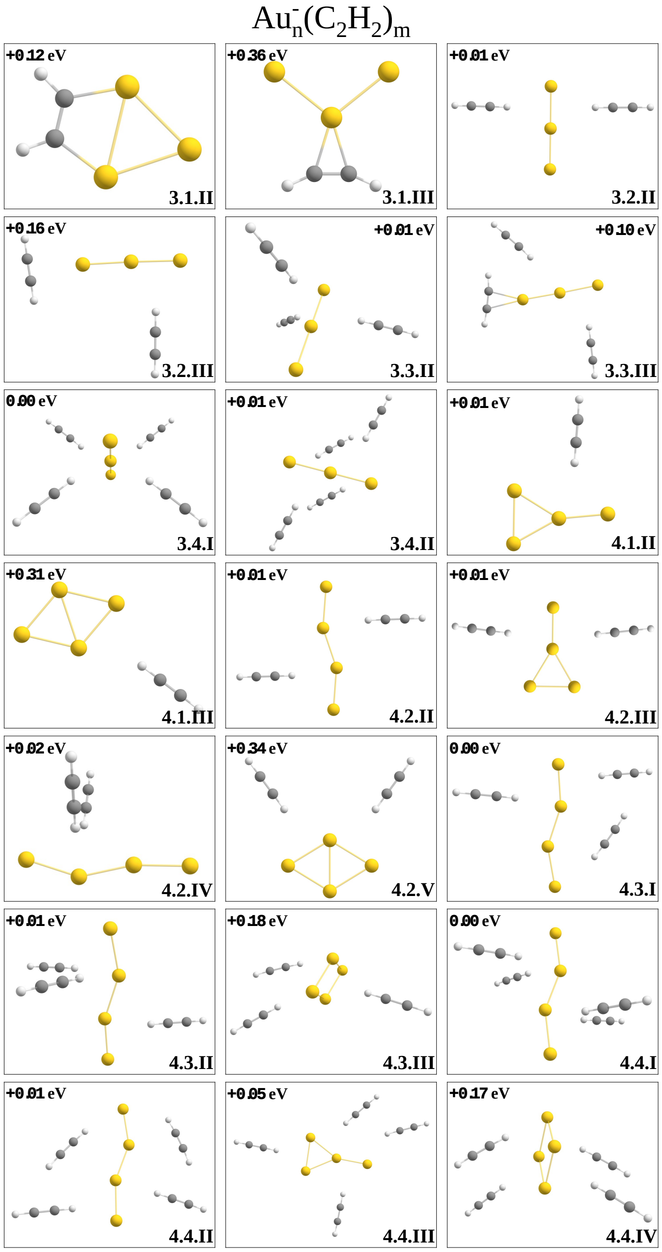

Figure S4: Alternative structures of $Au_n^-(C_2H_2)_m$, that where calculated during the computational process.

4 Natural bond order (NBO) analysis at the ωB97X-D/Def2TZVP level of theory

The following NBO analysis shows the natural electron configuration of cationic and anionic gold-acetylene complexes with respect to gold cluster growth and additional acetylene attachment. Orbital occupation numbers smaller than 0.05 were ignored.

4.1 Natural Electron Configuration of cations for increasing number of acetylene

$Au^+C_2H_2$ iso I

C	[core]$2s^{1.08}$ $2p^{3.14}$
H	$1s^{0.69}$
C	[core]$2s^{1.08}$ $2p^{3.14}$
Au	[core]$6s^{0.38}$ $5d^{9.76}$
H	$1s^{0.69}$

$Au^+(C_2H_2)_2$ iso I

Au	[core]$6s^{0.66}$ $5d^{9.55}$ $6p^{0.06}$
C	[core]$2s^{1.06}$ $2p^{3.16}$
H	$1s^{0.69}$
C	[core]$2s^{1.06}$ $2p^{3.16}$
H	$1s^{0.69}$
H	$1s^{0.69}$
C	[core]$2s^{1.06}$ $2p^{3.16}$
C	[core]$2s^{1.06}$ $2p^{3.16}$
H	$1s^{0.69}$

$Au^+(C_2H_2)_3$ iso I

Au	[core]$6s^{0.64}$ $5d^{9.56}$ $6p^{0.08}$
C	[core]$2s^{1.06}$ $2p^{3.16}$
C	[core]$2s^{1.05}$ $2p^{3.17}$
C	[core]$2s^{1.06}$ $2p^{3.16}$
C	[core]$2s^{1.05}$ $2p^{3.17}$
H	$1s^{0.69}$
H	$1s^{0.69}$
H	$1s^{0.69}$
H	$1s^{0.69}$
C	[core]$2s^{1.03}$ $2p^{3.20}$
C	[core]$2s^{1.03}$ $2p^{3.20}$
H	$1s^{0.75}$
H	$1s^{0.75}$

$Au^+(C_2H_2)_4$ iso I

Au	[core]$6s^{0.63}$ $5d^{9.56}$ $6p^{0.11}$
C	[core]$2s^{1.05}$ $2p^{3.17}$
C	[core]$2s^{1.05}$ $2p^{3.17}$
C	[core]$2s^{1.05}$ $2p^{3.17}$
C	[core]$2s^{1.05}$ $2p^{3.17}$
C	[core]$2s^{1.03}$ $2p^{3.20}$
C	[core]$2s^{1.03}$ $2p^{3.20}$
C	[core]$2s^{1.03}$ $2p^{3.20}$
C	[core]$2s^{1.03}$ $2p^{3.20}$
H	$1s^{0.69}$
H	$1s^{0.69}$
H	$1s^{0.69}$
H	$1s^{0.69}$
H	$1s^{0.75}$
H	$1s^{0.75}$
H	$1s^{0.75}$
H	$1s^{0.75}$

4.2 Natural Electron Configuration of cations for increasing Au cluster size

$Au^+C_2H_2$ iso I

C	[core]$2s^{1.08}$ $2p^{3.14}$
H	$1s^{0.69}$
C	[core]$2s^{1.08}$ $2p^{3.14}$
Au	[core]$6s^{0.38}$ $5d^{9.76}$
H	$1s^{0.69}$

$Au_2^+C_2H_2$ iso I

C	[core]$2s^{1.06}$ $2p^{3.16}$
C	[core]$2s^{1.06}$ $2p^{3.16}$
Au	[core]$6s^{0.72}$ $5d^{9.75}$ $6p^{0.10}$
H	$1s^{0.70}$
H	$1s^{0.70}$
Au	[core]$6s^{0.69}$ $5d^{9.86}$

$Au_3^+C_2H_2$ iso I

Au	[core]$6s^{0.78}$ $5d^{9.74}$ $6p^{0.19}$
Au	[core]$6s^{0.71}$ $5d^{9.93}$ $6p^{0.05}$
Au	[core]$6s^{0.71}$ $5d^{9.93}$ $6p^{0.05}$
C	[core]$2s^{1.05}$ $2p^{3.17}$
C	[core]$2s^{1.05}$ $2p^{3.17}$
H	$1s^{0.70}$
H	$1s^{0.70}$

$Au_4^+C_2H_2$ iso I

Au	[core]$6s^{0.72}$ $5d^{9.94}$
Au	[core]$6s^{0.72}$ $5d^{9.94}$
Au	[core]$6s^{0.83}$ $5d^{9.87}$ $6p^{0.13}$
Au	[core]$6s^{0.73}$ $5d^{9.74}$ $6p^{0.37}$
C	[core]$2s^{1.04}$ $2p^{3.19}$
C	[core]$2s^{1.04}$ $2p^{3.19}$
H	$1s^{0.70}$
H	$1s^{0.70}$

4.3 Natural Electron Configuration of anions for increasing number of acetylene

$Au^{-}C_2H_2$ iso I

Au	[core]$6s^{1.97}$ $5d^{9.99}$
H	$1s^{0.72}$
C	[core]$2s^{1.06}$ $2p^{3.11}$
C	[core]$2s^{1.01}$ $2p^{3.32}$
H	$1s^{0.78}$

$Au^{-}(C_2H_2)_2$ iso I

Au	[core]$6s^{1.96}$ $5d^{9.99}$
H	$1s^{0.72}$
H	$1s^{0.72}$
C	[core]$2s^{1.05}$ $2p^{3.12}$
C	[core]$2s^{1.01}$ $2p^{3.31}$
C	[core]$2s^{1.05}$ $2p^{3.12}$
C	[core]$2s^{1.01}$ $2p^{3.31}$
H	$1s^{0.78}$
H	$1s^{0.78}$

$Au^{-}(C_2H_2)_3$ iso I

Au	[core]$6s^{1.94}$ $5d^{9.98}$
H	$1s^{0.73}$
H	$1s^{0.73}$
C	[core]$2s^{1.05}$ $2p^{3.12}$
C	[core]$2s^{1.01}$ $2p^{3.31}$
C	[core]$2s^{1.05}$ $2p^{3.12}$
C	[core]$2s^{1.01}$ $2p^{3.31}$
H	$1s^{0.78}$
H	$1s^{0.78}$
H	$1s^{0.73}$
H	$1s^{0.78}$
C	[core]$2s^{1.01}$ $2p^{3.31}$
C	[core]$2s^{1.05}$ $2p^{3.12}$

$Au^{-}(C_2H_2)_4$ iso I

Au	[core]$6s^{1.93}$ $5d^{9.98}$
C	[core]$2s^{1.05}$ $2p^{3.13}$
C	[core]$2s^{1.01}$ $2p^{3.30}$
C	[core]$2s^{1.05}$ $2p^{3.13}$
C	[core]$2s^{1.01}$ $2p^{3.30}$
H	$1s^{0.78}$
H	$1s^{0.73}$
H	$1s^{0.78}$
H	$1s^{0.73}$
C	[core]$2s^{1.01}$ $2p^{3.30}$
C	[core]$2s^{1.05}$ $2p^{3.13}$
H	$1s^{0.78}$
H	$1s^{0.73}$
C	[core]$2s^{1.05}$ $2p^{3.13}$
C	[core]$2s^{1.01}$ $2p^{3.30}$
H	$1s^{0.73}$
H	$1s^{0.78}$

4.4 Natural Electron Configuration of anions for increasing Au cluster size

$Au^{-}C_2H_2$ iso I

Au	[core]$6s^{1.97}$ $5d^{9.99}$
H	$1s^{0.72}$
C	[core]$2s^{1.06}$ $2p^{3.11}$
C	[core]$2s^{1.01}$ $2p^{3.32}$
H	$1s^{0.78}$

$Au_2^{-}C_2H_2$ iso I

Au	[core]$6s^{1.48}$ $5d^{9.95}$ $6p^{0.06}$
Au	[core]$6s^{1.48}$ $5d^{9.95}$ $6p^{0.06}$
C	[core]$2s^{1.04}$ $2p^{3.13}$
C	[core]$2s^{1.01}$ $2p^{3.30}$
H	$1s^{0.78}$
H	$1s^{0.73}$

$Au_3^{-}C_2H_2$ iso I

Au	[core]$6s^{1.05}$ $5d^{9.91}$ $6p^{0.21}$
Au	[core]$6s^{1.46}$ $5d^{9.94}$
Au	[core]$6s^{1.45}$ $5d^{9.94}$
C	[core]$2s^{1.04}$ $2p^{3.14}$
C	[core]$2s^{1.01}$ $2p^{3.29}$
H	$1s^{0.74}$
H	$1s^{0.78}$

$Au_4^{-}C_2H_2$ iso I

Au	[core]$6s^{1.32}$ $5d^{9.95}$
Au	[core]$6s^{1.20}$ $5d^{9.91}$ $6p^{0.11}$
Au	[core]$6s^{1.35}$ $5d^{9.95}$
Au	[core]$6s^{1.15}$ $5d^{9.92}$ $6p^{0.10}$
C	[core]$2s^{1.03}$ $2p^{3.15}$
C	[core]$2s^{1.01}$ $2p^{3.28}$
H	$1s^{0.74}$
H	$1s^{0.78}$

5 CHELPG calculations

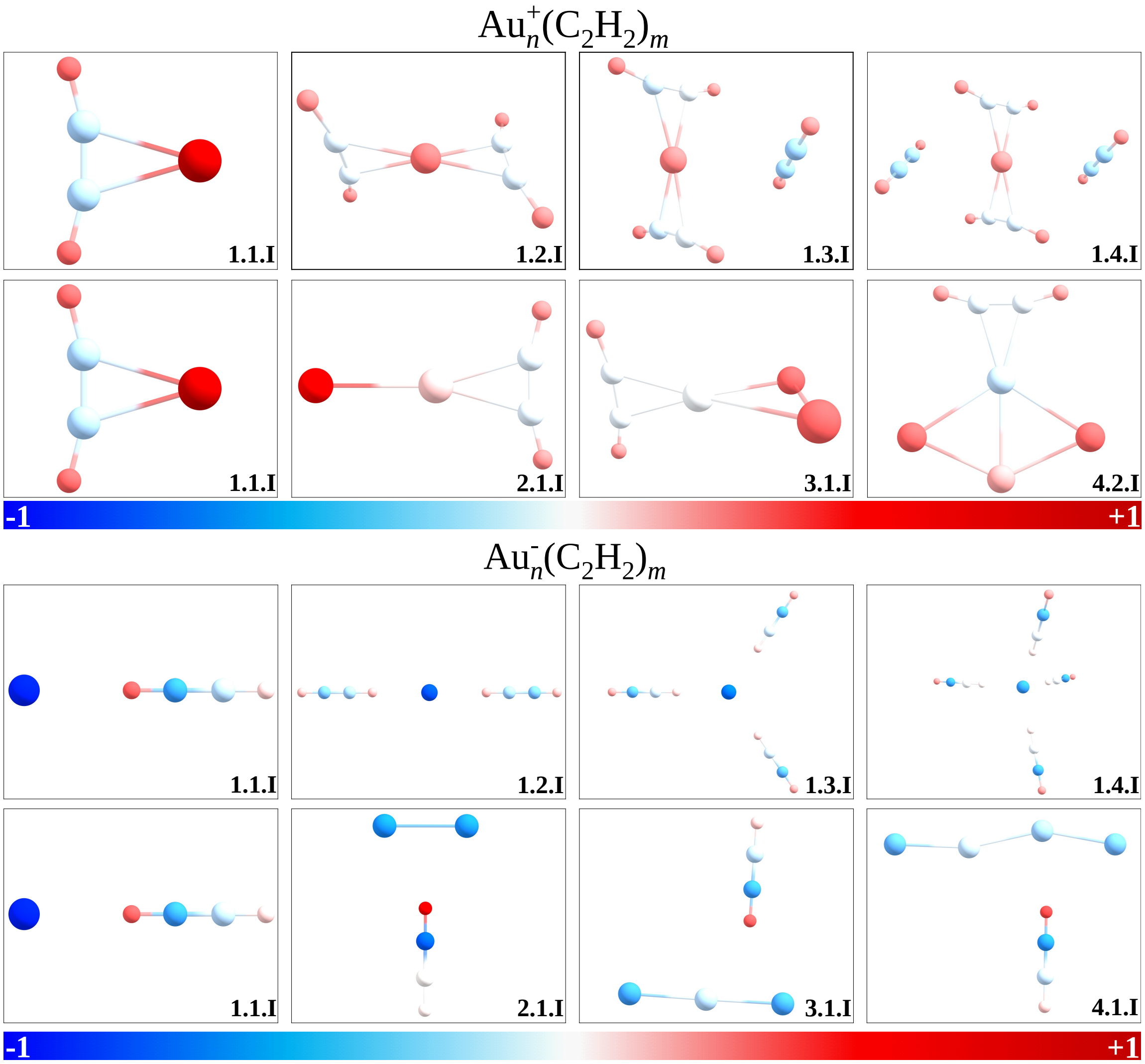

Figure S5: Electrostatic potential charges of $Au_n^{+/-}(C_2H_2)_m$, calculated by the CHELPG method. The charges are colour coded from $q = -1$ (blue) over $q = 0$ (white) to $q = 1$ (red).

6 Energy Decomposition Analysis

The energy decomposition analysis was done using the EDA-NOCV analysis implemented within ORCA [1–6]. The EDA-NOCV analysis was performed for cationic and anionic gold-acetylene complexes with respect to the growth of the gold cluster and the addition of acetylene. The ωB97x-d3 functional was used to perform these calculations, due to the unavailability of the ωB97x-d functional in ORCA.

6.1 Energy Decomposition Analysis of cations for increasing number of acetylene

$Au^+C_2H_2$ iso I

Energy Component	Energy (Hartree)	Energy (eV)	Energy (kcal/mol)
Bond Energy	-0.0899585832	-2.45	-56.45
Orbital Energy	-0.1517037680	-4.13	-95.20
Electrostatic Energy	-0.1867665548	-5.08	-117.20
Pauli Energy	0.3256333410	8.87	204.34
ΔE^0(XC)	-0.0767990347	-2.09	-48.19
Δ Dispersion	-0.0002839790	-0.01	-0.18

$Au^+(C_2H_2)_2$ iso I

Energy Component	Energy (Hartree)	Energy (eV)	Energy (kcal/mol)
Bond Energy	-0.0879941502	-2.39	-55.22
Orbital Energy	-0.1372315366	-3.73	-86.11
Electrostatic Energy	-0.1665941407	-4.53	-104.54
Pauli Energy	0.2883600020	7.85	180.95
ΔE^0(XC)	-0.0718753053	-1.96	-45.10
Δ Dispersion	-0.0008113115	-0.02	-0.51

$Au^+(C_2H_2)_3$ iso I

Energy Component	Energy (Hartree)	Energy (eV)	Energy (kcal/mol)
Bond Energy	-0.0104162015	-0.28	-6.54
Orbital Energy	-0.0052017334	-0.14	-3.26
Electrostatic Energy	-0.0192487422	-0.52	-12.08
Pauli Energy	0.0242950630	0.66	15.25
ΔE^0(XC)	-0.0076607415	-0.21	-4.81
Δ Dispersion	-0.0025942882	-0.07	-1.63

$Au^+(C_2H_2)_4$ iso I

Energy Component	Energy (Hartree)	Energy (eV)	Energy (kcal/mol)
Bond Energy	-0.0095310727	-0.26	-5.98
Orbital Energy	-0.0042130781	-0.11	-2.64
Electrostatic Energy	-0.0169588398	-0.46	-10.64
Pauli Energy	0.0211399633	0.58	13.27
ΔE^0(XC)	-0.0068086395	-0.19	-4.27
Δ Dispersion	-0.0026639063	-0.07	-1.67

6.2 Energy Decomposition Analysis of cations for increasing Au cluster size

$Au^+C_2H_2$ iso I

Energy Component	Energy (Hartree)	Energy (eV)	Energy (kcal/mol)
Bond Energy	-0.0899585832	-2.45	-56.45
Orbital Energy	-0.1517037680	-4.13	-95.20
Electrostatic Energy	-0.1867665548	-5.08	-117.20
Pauli Energy	0.3256333410	8.87	204.34
ΔE^0(XC)	-0.0767990347	-2.09	-48.19
Δ Dispersion	-0.0002839790	-0.01	-0.18

$Au_2^+C_2H_2$ iso I

Energy Component	Energy (Hartree)	Energy (eV)	Energy (kcal/mol)
Bond Energy	-0.0711974679	-1.94	-44.68
Orbital Energy	-0.1330347462	-3.62	-83.48
Electrostatic Energy	-0.1860440498	-5.06	-116.74
Pauli Energy	0.3300140135	8.98	207.09
ΔE^0(XC)	-0.0763438235	-2.08	-47.91
Δ Dispersion	-0.0007819410	-0.02	-0.49

$Au_3^+C_2H_2$ iso I

Energy Component	Energy (Hartree)	Energy (eV)	Energy (kcal/mol)
Bond Energy	-0.0647830837	-1.76	-40.65
Orbital Energy	-0.1204519190	-3.28	-75.58
Electrostatic Energy	-0.1735017274	-4.72	-108.87
Pauli Energy	0.3029943631	8.25	190.13
ΔE^0(XC)	-0.0724173676	-1.97	-45.44
Δ Dispersion	-0.0013615629	-0.04	-0.85

$Au_4^+C_2H_2$ iso I

Energy Component	Energy (Hartree)	Energy (eV)	Energy (kcal/mol)
Bond Energy	-0.0614886082	-1.67	-38.58
Orbital Energy	-0.1272467751	-3.46	-79.85
Electrostatic Energy	-0.1883733156	-5.13	-118.21
Pauli Energy	0.3345931820	9.10	209.96
ΔE^0(XC)	-0.0780678502	-2.13	-48.99
Δ Dispersion	-0.0023508664	-0.06	-1.48

6.3 Energy Decomposition Analysis of anions for increasing number of acetylene

$Au^-C_2H_2$ iso I

Energy Component	Energy (Hartree)	Energy (eV)	Energy (kcal/mol)
Bond Energy	-0.0117083113	-0.32	-7.35
Orbital Energy	-0.0088704177	-0.24	-5.57
Electrostatic Energy	-0.0284077601	-0.77	-17.83
Pauli Energy	0.0342010086	0.93	21.46
ΔE^0(XC)	-0.0079562877	-0.22	-4.99
Δ Dispersion	-0.0006766698	-0.02	-0.42

$Au^-(C_2H_2)_2$ iso I

Energy Component	Energy (Hartree)	Energy (eV)	Energy (kcal/mol)
Bond Energy	-0.0104719917	-0.28	-6.57
Orbital Energy	-0.0075540841	-0.21	-4.74
Electrostatic Energy	-0.0246082964	-0.67	-15.44
Pauli Energy	0.0294104718	0.80	18.46
ΔE^0(XC)	-0.0070184187	-0.19	-4.40
Δ Dispersion	-0.0007014180	-0.02	-0.44

$Au^-(C_2H_2)_3$ iso I

Energy Component	Energy (Hartree)	Energy (eV)	Energy (kcal/mol)
Bond Energy	-0.0098618203	-0.27	-6.19
Orbital Energy	-0.0067991911	-0.18	-4.27
Electrostatic Energy	-0.0223824461	-0.61	-14.05
Pauli Energy	0.0265200822	0.72	16.64
ΔE^0(XC)	-0.0064270195	-0.17	-4.03
Δ Dispersion	-0.0007709568	-0.02	-0.48

$Au^-(C_2H_2)_4$ iso I

**Energy Component**	**Energy (Hartree)**	**Energy (eV)**	**Energy (kcal/mol)**
Bond Energy	-0.0092768941	-0.25	-5.82
Orbital Energy	-0.0061707470	-0.17	-3.87
Electrostatic Energy	-0.0205665690	-0.56	-12.91
Pauli Energy	0.0243443765	0.66	15.28
ΔE^0(XC)	-0.0059872628	-0.16	-3.76
Δ Dispersion	-0.0008895151	-0.02	-0.56

6.4 Energy Decomposition Analysis of anions for for increasing Au cluster size

$Au^-C_2H_2$ iso I

**Energy Component**	**Energy (Hartree)**	**Energy (eV)**	**Energy (kcal/mol)**
Bond Energy	-0.0117083113	-0.32	-7.35
Orbital Energy	-0.0088704177	-0.24	-5.57
Electrostatic Energy	-0.0284077601	-0.77	-17.83
Pauli Energy	0.0342010086	0.93	21.46
ΔE^0(XC)	-0.0079562877	-0.22	-4.99
Δ Dispersion	-0.0006766698	-0.02	-0.42

$Au_2^-C_2H_2$ iso I

**Energy Component**	**Energy (Hartree)**	**Energy (eV)**	**Energy (kcal/mol)**
Bond Energy	-0.0092038599	-0.25	-5.78
Orbital Energy	-0.0046255493	-0.13	-2.90
Electrostatic Energy	-0.0159221139	-0.43	-9.99
Pauli Energy	0.0181990753	0.50	11.42
ΔE^0(XC)	-0.0051171717	-0.14	-3.21
Δ Dispersion	-0.0014710046	-0.04	-0.92

$Au_3^-C_2H_2$ iso I

**Energy Component**	**Energy (Hartree)**	**Energy (eV)**	**Energy (kcal/mol)**
Bond Energy	-0.0077480682	-0.21	-4.86
Orbital Energy	-0.0039659668	-0.11	-2.49
Electrostatic Energy	-0.0131884215	-0.36	-8.28
Pauli Energy	0.0156839625	0.43	9.84
ΔE^0(XC)	-0.0044676963	-0.12	-2.80
Δ Dispersion	-0.0017054119	-0.05	-1.07

$Au_4^-C_2H_2$ iso I

**Energy Component**	**Energy (Hartree)**	**Energy (eV)**	**Energy (kcal/mol)**
Bond Energy	-0.0067985938	-0.18	-4.27
Orbital Energy	-0.0034162513	-0.09	-2.14
Electrostatic Energy	-0.0117778107	-0.32	-7.39
Pauli Energy	0.0154508980	0.42	9.70
ΔE^0(XC)	-0.0046587141	-0.13	-2.92
Δ Dispersion	-0.0022697940	-0.06	-1.42

References

[1] F. Neese, *WIRES Comput. Mol. Sci.*, 2025, **15**, e70019.

[2] F. Neese, *J. Comput. Chem.*, 2003, **24**, 1740–1747.

[3] F. Neese, F. Wennmohs, A. Hansen and U. Becker, *Chem. Phys.*, 2009, **356**, 98–109.

[4] S. Grimme, J. Antony, S. Ehrlich and H. Krieg, *J. Chem. Phys.*, 2010, **132**, 154104.

[5] B. Helmich-Paris, B. de Souza, F. Neese and R. Izsák, *J. Chem. Phys.*, 2021, **155**, 104109.

[6] F. Neese, *J. Comput. Chem.*, 2022, **44**, 381.
